# Efficacy and safety of efruxifermin for patients with NASH/MASH: an updated systematic review and meta-analysis

**DOI:** 10.3389/fphar.2025.1731065

**Published:** 2026-04-02

**Authors:** Ya-Jun Xiao, Xue-Ping Liu, Yan-Ling Zhang, Yan Cheng, Xiao-Li Tian, Yan-Qun Liu, Cun-Liang Deng, Hao Sun

**Affiliations:** 1 Department of Geriatrics, The Third Hospital of Mianyang, Sichuan Mental Health Center, Mianyang, Sichuan, China; 2 Department of Infectious Diseases, The Affiliated Hospital, Southwest Medical University, Luzhou, Sichuan, China; 3 Department of Dermatology, The Affiliated Hospital, Southwest Medical University, Luzhou, Sichuan, China; 4 Dalian Medical University, Dalian, Liaoning, China; 5 Department of Nephrology, The Third Hospital of Mianyang, Sichuan Mental Health Center, Mianyang, Sichuan, China; 6 Department of Nephrology, The Affiliated Hospital, Southwest Medical University, Luzhou, Sichuan, China; 7 Department of Medical Equipment Management, The Third Hospital of Mianyang, Sichuan Mental Health Center, Mianyang, Sichuan, China

**Keywords:** efruxifermin, NASH, MASH, fibrosis, steatohepatitis, meta-analysis

## Abstract

**Aims:**

Efruxifermin is a promising treatment for non-alcoholic steatohepatitis (NASH), now referred to as metabolic dysfunction-associated steatohepatitis (MASH). This meta-analysis aims to evaluate the efficacy and safety of efruxifermin in patients with NASH/MASH.

**Methods:**

We systematically searched PubMed, Embase, the Cochrane Library, and ClinicalTrials.gov for randomized controlled trials (RCTs) evaluating the efficacy and safety of efruxifermin in patients with NASH/MASH up to 6 August 2025. The primary outcomes were changes in liver fibrosis and steatosis, with safety assessed through adverse events.

**Results:**

This meta-analysis included 4 RCTs with 419 participants. Compared with placebo, efruxifermin demonstrated a significant advantage in ≥1 stage improvement in liver fibrosis without worsening steatohepatitis (relative risk [RR]: 2.18, 95% confidence interval [CI] [1.34, 3.57], P = 0.002), NASH/MASH resolution with fibrosis improvement (RR: 5.15, 95% CI [1.52, 17.47], P = 0.009), and ≥2-point non-alcoholic fatty liver disease activity score (NAS) improvement without fibrosis worsening (RR: 3.34, 95% CI [1.93, 5.80], P < 0.001). Additionally, efruxifermin reduced the enhanced liver fibrosis (ELF) score, liver stiffness measurement (LSM), and serum levels of N-terminal type-III collagen pro-peptide (ProC3). For steatosis reduction, efruxifermin significantly increased the proportions of patients with ≥30% hepatic fat fraction (HFF) reduction (RR: 4.69, 95% CI [2.53, 8.71], P < 0.001), ≥50% HFF reduction (RR: 22.57, 95% CI [5.78, 88.22], P < 0.001), and liver fat normalization (RR: 13.03, 95% CI [3.30, 51.50], P < 0.001). However, efruxifermin treatment was associated with higher rates of both adverse events leading to discontinuation and gastrointestinal adverse events.

**Conclusion:**

Efruxifermin may represent a promising therapeutic option for NASH/MASH. Given the limitations in both the number and short follow-up duration of the included RCTs, the conclusions should be interpreted with caution. Further large-scale, multicenter, long-term, and high-quality RCTs are necessary to validate these results in diverse populations.

**Systematic Review Registration:**

https://www.crd.york.ac.uk/PROSPERO/, identifier CRD42025111 4840.

## 1 Introduction

Metabolic dysfunction-associated fatty liver disease (MASLD), previously known as non-alcoholic fatty liver disease (NAFLD), is a highly prevalent chronic liver disease, affecting approximately 33% of the global population (Fouad et al., 2024). Its severe phenotype, metabolic dysfunction-associated steatohepatitis (MASH), formerly referred to as non-alcoholic steatohepatitis (NASH) (Rinella et al., 2023b), is characterized by hepatic steatosis and inflammation, with the potential to progress to cirrhosis (Hagström et al., 2024).

The pathogenesis and progression of MASH are complex. Within hepatocytes, lipotoxicity and the associated oxidative stress can induce endoplasmic reticulum stress, activate pro-apoptotic pathways, and trigger the release of inflammatory mediators, thereby promoting hepatic inflammation, hepatocyte death, and the progression of liver fibrosis (Lebeaupin et al., 2018). Fibrosis occurs in response to chronic injury and inflammation. Left unaddressed, NASH fibrosis may advance to cirrhosis, which in turn may cause end-stage liver disease or hepatocellular carcinoma (HCC) (Tillman and Rolph, 2020). Patients with cirrhosis caused by NASH/MASH have a poor prognosis, with a significantly increased risk of hepatic decompensation, HCC and mortality (Hagström et al., 2024; Sanyal et al., 2021). Therefore, effectively intervening in the processes of hepatic steatosis and fibrosis is crucial for halting disease progression and preventing fatal complications such as cirrhosis, liver failure, and HCC.

Fibroblast growth factor 21 (FGF21) is an endocrine member of the FGF19 subfamily (Tillman and Rolph, 2020), functioning as a hormone that regulates glucose and lipid metabolism, insulin sensitivity, and protein homeostasis (Harrison et al., 2024a). FGF21 activates the membrane coreceptor complex of β-klotho and one of its homologous fibroblast growth factor receptors (FGFRs), including FGFR1c, FGFR2c, or FGFR3c. By acting directly or indirectly on multiple major organs, particularly adipose tissue, the liver, and the brain, FGF21 provides protection against obesity, insulin resistance, and disorders of vascular homeostasis.

FGF21 analogues have shown significant potential in the treatment of MASH. Their mechanism of action aligns with several core characteristics of an ideal therapeutic strategy: they not only target the primary pathological driver of excessive hepatic fat accumulation (Camporez et al., 2013; Xu et al., 2009) but also modulate the subsequent inflammatory and fibrotic cascades (Ji et al., 2024; Xu et al., 2016; Meng et al., 2021). FGF21 inhibits pro-fibrotic signaling pathways through both direct and indirect mechanisms (Tillman and Rolph, 2020). FGF21 regulates fatty acid activation and oxidation in livers of mice. In the absence of FGF21, accumulation of inactivated fatty acids results in lipotoxic damage and increased steatosis (Fisher et al., 2014). The direct mechanism is characterized by the inhibition of the transformation of hepatic stellate cells (HSCs) into collagen-secreting myofibroblasts (Tillman and Rolph, 2020).

Efruxifermin is a long-acting human immunoglobulin 1 (IgG1) Fc-FGF21 fusion protein. Its C-terminal region has been modified with two amino acid substitutions (P171G and A180E). Optimizing the design significantly extends its pharmacokinetic (PK) and pharmacodynamic (PD) half-lives, thus facilitating a once-weekly dosing regimen (Kaufman et al., 2020). Furthermore, efruxifermin enhances binding affinity to the essential co-receptor β-Klotho. It exhibits balanced *in vitro* potency towards the FGF21 receptors FGFR1c, FGFR2c, and FGFR3c, and demonstrates high systemic exposure *in vivo* (Kaufman et al., 2020), allowing it to effectively target adipose tissue and the liver.

In a Phase 2b trial involving patients with stage 2 or 3 liver fibrosis due to NASH, efruxifermin demonstrated a significant reduction in the degree of liver fibrosis and alleviation of NASH (Harrison et al., 2023). However, a Phase 2b clinical trial published in May 2025 indicated that efruxifermin did not achieve a significant reversal of fibrosis without exacerbating MASH in patients with compensated cirrhosis (F4) caused by MASH at 36 weeks; potential benefits were observed at 96 weeks (Noureddin et al., 2025). To date, only one systematic review and meta-analysis (Li et al., 2025) has been conducted on efruxifermin; however, this study did not include the results reported in the aforementioned latest randomized controlled trial (RCT), nor did it integrate key outcome measures such as liver fat fraction (HFF). Therefore, this study aims to conduct an updated and more comprehensive systematic review and meta-analysis to systematically evaluate the efficacy and safety of efruxifermin in patients with NASH/MASH, thereby providing a solid evidence-based medical foundation for subsequent clinical research.

## 2 Methods

This systematic review was conducted in accordance with the Cochrane Handbook for Systematic Reviews of Interventions (Higgins et al., 2024) and was reported following the guidelines of the Preferred Reporting Items for Systematic Reviews and Meta-Analyses (PRISMA) (Page et al., 2021). The study protocol was prospectively registered on the International Prospective Register of Systematic Reviews (PROSPERO) before data extraction (Registration number: CRD42025111 4840).

### 2.1 Data sources and searches

An extensive search for RCTs was conducted in PubMed, Embase, the Cochrane Library, and ClinicalTrials.gov from inception through Aug 6, 2025. Medical Subject Headings (MeSH) or the keywords “Non-alcoholic steatohepatitis”, “Metabolic dysfunction-associated steatohepatitis”, and “efruxifermin” were used to search the literature without language restrictions. The PubMed search strategy was detailed in Supplementary Table S1. Additionally, the references of included studies were reviewed to ensure comprehensive coverage of the relevant literature.

### 2.2 Study selection

Two independent reviewers, Ya-Jun Xiao and Xue-Ping Liu, conducted a comprehensive review of the articles by evaluating the title, abstract, and full text. After removing duplicates, studies were selected based on the following Population, Intervention, Comparison, Outcomes, and Study design (PICOS) criteria: The population (P) consisted of participants aged 18 years or older diagnosed with NASH/MASH by biopsy; the intervention (I) was administration of efruxifermin, including different dosing regimens (28 mg/week, 50 mg/week, and 70 mg/week, all administered as once-weekly subcutaneous injections); the comparison (C) was placebo or active comparator; the outcomes (O) included at least one of the pre-specified outcomes of interest; and the study design (S) employed a RCT design. The exclusion criteria were as follows: (1) non-RCTs; (2) study protocols, ongoing trials with unavailable results, letters, reviews, conference abstracts, and meta-analyses; (3) studies not involving efruxifermin; (4) studies not related to NASH/MASH; (5) studies with overlapping populations or duplicate publications; and (6) non-human studies. Any discrepancies were resolved through discussion; if necessary, a third reviewer, was consulted.

The primary outcome was the proportion of patients exhibiting an improvement in liver fibrosis by one or more stages without any worsening of steatohepatitis. Worsening was defined as an increase in the score for any component of the NAFLD Activity Score (NAS), specifically ballooning, inflammation, or steatosis. Additionally, the study assessed the proportion of patients achieving resolution of NASH/MASH alongside fibrosis improvement, as well as those attaining a reduction of ≥2 points in NAS without worsening of the fibrosis stage. Secondary outcomes included the enhanced liver fibrosis (ELF) score, liver stiffness measurement (LSM), and N-terminal type-III collagen pro-peptide (ProC3) levels. Also included in the secondary outcomes were reduction in hepatic steatosis and the proportion of patients achieving liver fat normalization. Safety outcomes included treatment-emergent adverse events (TEAEs), serious AEs, AEs leading to treatment discontinuation, and those occurring in ≥15% of participants.

### 2.3 Data extraction

The reference management software EndNote 20 was utilized to organize all studies that were retrieved. Details from the eligible studies were independently extracted by two researchers. This included information such as the first author’s name, publication year, primary and secondary outcomes, dosage of experimental drug, study duration, and basic characteristics of patients (such as the number of participants, mean age, gender, race or ethnicity, metabolic risk factors and parameters, liver histology, and markers of fibrosis).

### 2.4 Quality assessment

The methodological quality of the included RCTs was assessed using the Cochrane Risk of Bias tool. This evaluation encompassed several domains, including the generation of random sequences, allocation concealment, blinding of participants and personnel, blinding of outcome assessment, Incomplete outcome data, selective reporting, and other potential sources of bias. Two authors independently evaluated each study across these domains, categorizing the risk of bias for each domain as low, high, or unclear. Any disagreements were resolved through consultation with a third author, to achieve consensus. The quality assessment results of the ROB tool for each study were visualized using the Review Manager 5.4 software.

The overall certainty of evidence for each primary and secondary outcome was independently assessed by two reviewers (Ya-Jun Xiao and Xue-Ping Liu) using the Grading of Recommendations, Assessment, Development, and Evaluations (GRADE) approach. The assessment was based on five domains: risk of bias, imprecision, inconsistency, indirectness, and publication bias. Evidence was categorized into one of four quality levels: high, moderate, low, or very low. Disagreements were resolved by consensus or, when necessary, by adjudication from a third reviewer.

### 2.5 Data synthesis and analysis

Data analyses were performed using RevMan 5.4 and Stata 17.0. For continuous and dichotomous outcomes, the weighted mean difference (WMD) and risk ratio (RR) with their 95% confidence intervals (CIs) were calculated, respectively. A random-effects model using the Mantel-Haenszel method was employed for all meta-analyses. This model was chosen *a priori* due to anticipated clinical and methodological heterogeneity among included studies, which may arise from variations in population baseline characteristics (e.g., age, disease severity), intervention details (e.g., dosage, duration), and study design considerations. Heterogeneity was quantified using the I^2^ statistic. I^2^ values of ≤25%, 26%–50%, and >50% were considered to indicate low, moderate, and high heterogeneity, respectively. A two-sided P-value of <0.05 was considered statistically significant. Subgroup analyses were not performed owing to the small number of included studies.

To evaluate the robustness of the meta-analysis results, this study conducted sensitivity analysis. Specifically, two strategies were employed: first, by altering the pooled effect size model through cross-validation between fixed-effect and random-effect models; second, by sequentially excluding individual studies for iterative validation. Given the limited number of studies included for secondary outcomes, sensitivity analyses were performed solely for primary outcomes in this study.

## 3 Results

### 3.1 Study selection

**FIGURE 1:**
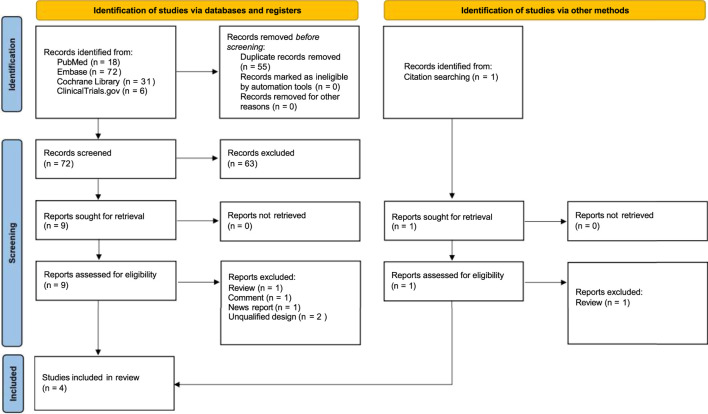
PRISMA flow diagram including reasons for exclusion of full-text articles.

As illustrated in Figure 1, a total of 122 articles and 6 clinical trials were initially identified. Upon eliminating duplicate entries and evaluating the studies by their titles and abstracts, 10 full-text articles were selected for comprehensive evaluation according to the predefined criteria. Ultimately, four RCTs (Harrison et al., 2023; Noureddin et al., 2025; Harrison et al., 2021a; Harrison et al., 2022) that met the inclusion and exclusion criteria were selected for our meta-analysis.

### 3.2 Study characteristics

We included four RCTs with a total of 419 participants. The baseline characteristics of the included trials were summarized in Table 1, with further details provided in Supplementary Table S2. The evaluated doses of efruxifermin were 28 mg, 50 mg, and 70 mg, administered subcutaneously once weekly. The follow-up duration ranged from 16 to 96 weeks. The four RCTs were conducted in the following locations: two exclusively in the United States, (Harrison et al., 2023; Harrison et al., 2022), one across the United States, Puerto Rico, and Mexico, (Noureddin et al., 2025), and one in both the United States and Puerto Rico (Harrison et al., 2021a). Regarding the study design, two of the included trials were Phase 2a clinical trials, (Harrison et al., 2021a; Harrison et al., 2022), while the other two were Phase 2b clinical trial (Harrison et al., 2023; Noureddin et al., 2025). All studies focused on patients with biopsy-proven NASH/MASH. Two studies exclusively recruited patients with compensated cirrhosis (F4), (Noureddin et al., 2025; Harrison et al., 2022), while one study (Harrison et al., 2021a) included patients with F1-F3 and another targeted patients with F2-F3 (Harrison et al., 2023). The majority of participants in the included studies were female. The mean age, body weight, BMI, as analyzed from the available patient data, were 57.2 years, 101.7 kg, 36.9 kg/m^2^, respectively.

**TABLE 1:** PICOS and baseline characteristics of trials included in the meta-analysis. Age and BMI were reported in Mean±SD.

Study	NCT ID	Population	Intervention	Comparison	Primary Outcomes	Study design	Follow-up	Age, years	Male, n (%)	BMI, kg/m^2^
Harrison et al., 2021a	NCT03976401	NASH (F1-F3)	Efruxifermin,28-50-70mg/once-weekly	Placebo	Absolute reduction in HFF at week 12	RCT, 2a	16 weeks	52.1 (12.2)	34 (43)	37.6 (6.7)
Harrison et al., 2022	NCT03976401	NASH (F4)	Efruxifermin, 50mg/once-weekly	Placebo	Safety and tolerability of efruxifermin	RCT, 2a	16 weeks	59.8 (11.6)	11 (37)	37.0 (6.6)
Harrison et al., 2023	NCT04767529	NASH (F2-F3)	Efruxifermin, 28-50mg/once-weekly	Placebo	Fibrosis improvement ≥ 1 stage without NASH worsening	RCT, 2b	24 weeks	54.7 (10.4)	49 (38)	38·0 (7·0)
Noureddin et al., 2025	NCT05039450	MASH (F4)	Efruxifermin, 28-50mg/once-weekly	Placebo	A reduction of at least one stage of fibrosis without worsening of MASH at week 36	RCT, 2b	96 weeks	60.7 (8.2)	60 (33)	35.8 (6.6)

Abbreviations: BMI, body mass index; HFF, hepatic fat fraction; MASH, metabolic dysfunction–associated steatohepatitis; NASH, non-alcoholic steatohepatitis; RCT, randomized controlled trial.

### 3.3 Risk of bias

**FIGURE 2:**
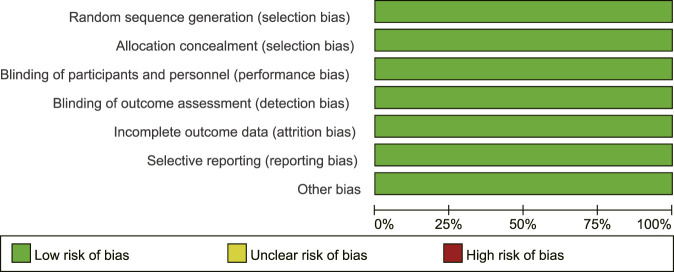
Risk of bias graph review authors' judgements.

**FIGURE 3 F3:**
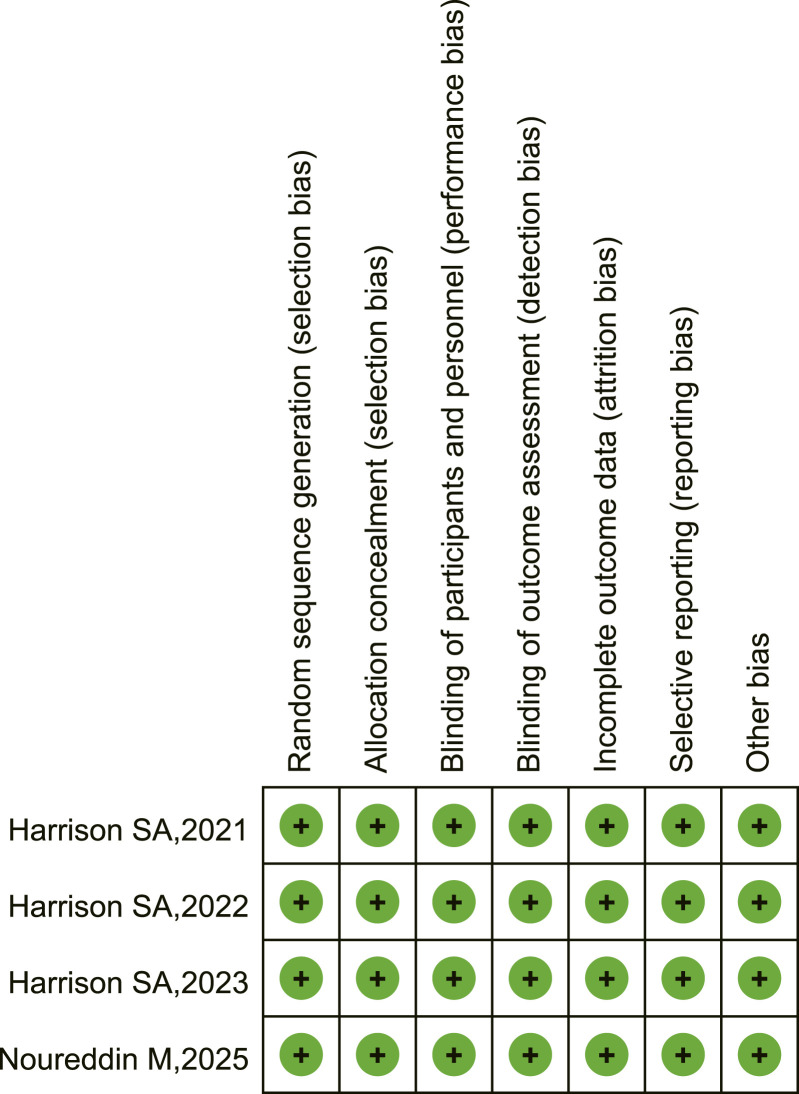
Risk of bias summary: review authors’ judgments about each risk of bias item for each included study.

The evaluation of bias risk for all included RCTs is presented in Figures 2, 3 [Fig F3]. Although Harrison et al. (2021) experienced a 16% rate of missing biopsy data at the end of the trial, the study stated that this was caused by the COVID-19 pandemic and did not affect the primary outcomes. Meanwhile, for missing values of the primary efficacy endpoints, the researchers employed multiple imputation methods; therefore, we consider the attrition bias to be at low risk observed attrition bias. The included studies were assessed as having a low risk of bias across all evaluated domains.

### 3.4 Results

#### 3.4.1 Histological assessment of liver biopsy

**FIGURE 4:**
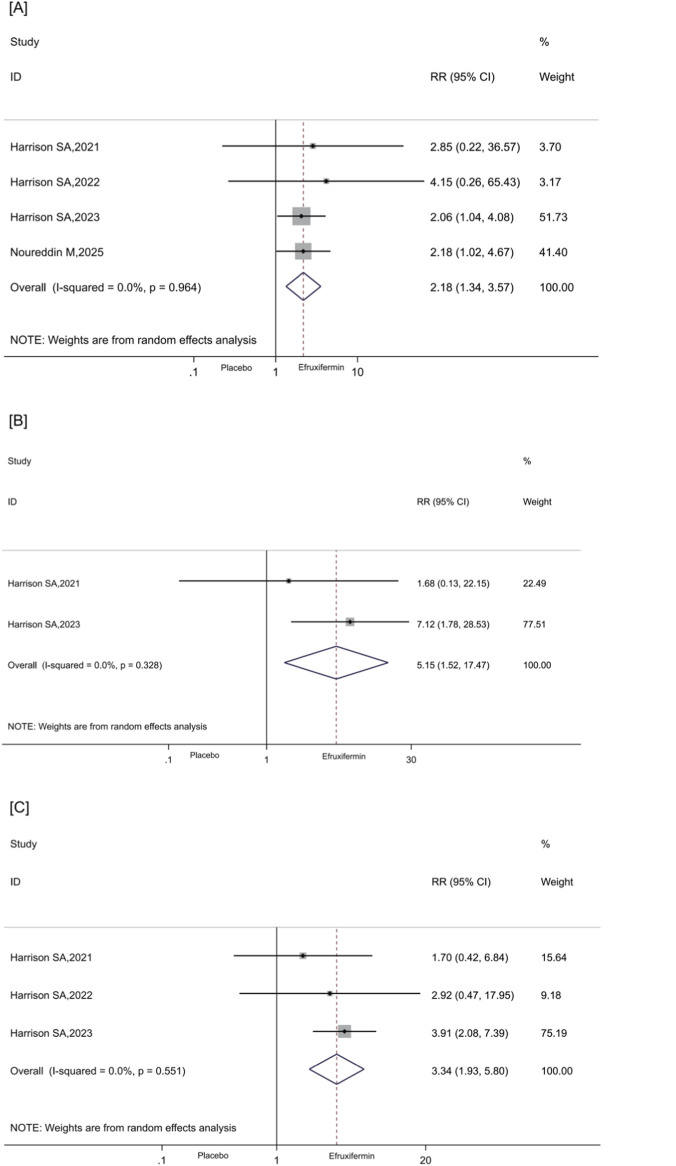
**(A–C)**. Forest plots for the meta-analysis comparing the effects of efruxifermin versus placebo on histological outcomes: **(A)** fibrosis regression by ≥1 stage and no worsening in steatohepatitis; **(B)** NASH/MASH resolution and improvement in fibrosis stage; **(C)** decrease in NAS ≥2 with no worsening in fibrosis stage. NASH, Non-alcoholic steatohepatitis; MASH, Metabolic dysfunction-associated steatohepatitis; NAS, Non-alcoholic fatty liver disease activity score; RR, relative risk; CI, confidence interval.

All included RCTs assessed efruxifermin *versus* placebo for ≥1-stage fibrosis regression and no steatohepatitis worsening; results showed a higher proportion of participants met this endpoint with efruxifermin (RR: 2.18, 95% CI [1.34, 3.57], P = 0.002, I^2^ = 0.0%, Figure 4A). Compared to placebo, efruxifermin demonstrated a significant advantage in achieving NASH/MASH resolution and improvement in fibrosis stage (RR: 5.15, 95% CI [1.52, 17.47], P = 0.009, I^2^ = 0.0%, Figure 4B). Additionally, it enhanced the decrease in NAS by ≥2 without worsening the fibrosis stage (RR: 3.34, 95% CI [1.93, 5.80], P < 0.001, I^2^ = 0.0%, Figure 4C). However, compared to placebo, efruxifermin did not demonstrate a significant advantage in promoting fibrosis regression by ≥2 stages and no worsening in steatohepatitis (RR: 2.58, 95% CI [0.72, 9.18], P = 0.144, I^2^ = 0.0%, Supplementary Figure S1).

#### 3.4.2 Non-invasive markers of fibrosis

**FIGURE 5:**
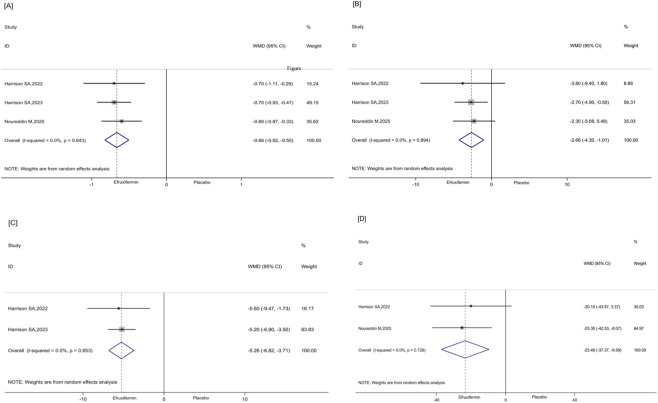
**(A–D)**. Forest plots for the meta-analysis comparing the effects of efruxifermin versus placebo on non-invasive markers of fibrosis: **(A)** Least-squares mean of the ELF test score; **(B)** Least-squares mean of LSM; **(C)** Least-squares mean absolute change in Pro-C3; **(D)** Least-squares mean percentage change in Pro-C3. ELF, enhanced liver fibrosis; LSM, liver stiffness measurement; ProC3, N-terminal type-III collagen pro-peptide; WMD, weighted mean difference; Cl, confidence interval.

Three RCTs (Harrison et al., 2023; Noureddin et al., 2025; Harrison et al., 2022) evaluated the effects of efruxifermin versus placebo on the least-squares mean (LS mean) values for the ELF test score and for LSM. The results indicated that efruxifermin treatment was associated with a reduction in the LS mean ELF test score (WMD: -0.66, 95% CI [-0.82, −0.50], P < 0.001, I^2^ = 0.0%, Figure 5A) and in LSM (WMD: −2.66 kPa, 95% CI [-4.3, −1.01], P < 0.001, I^2^ = 0.0%, Figure 5B). Two trials evaluated the effects of efruxifermin versus placebo on Pro-C3 levels. Efruxifermin was associated with a significant reduction in both the LS mean absolute change in Pro-C3 (WMD: -5.26 μg/L, 95% CI [-6.82, −3.71], P < 0.001, I^2^ = 0.0%, Figure 5C) and the LS mean percentage change (WMD: -23.48%, 95% CI [-37.37, −9.59], P = 0.001, I^2^ = 0.0%, Figure 5D).

#### 3.4.3 Change in HFF

**FIGURE 6:**
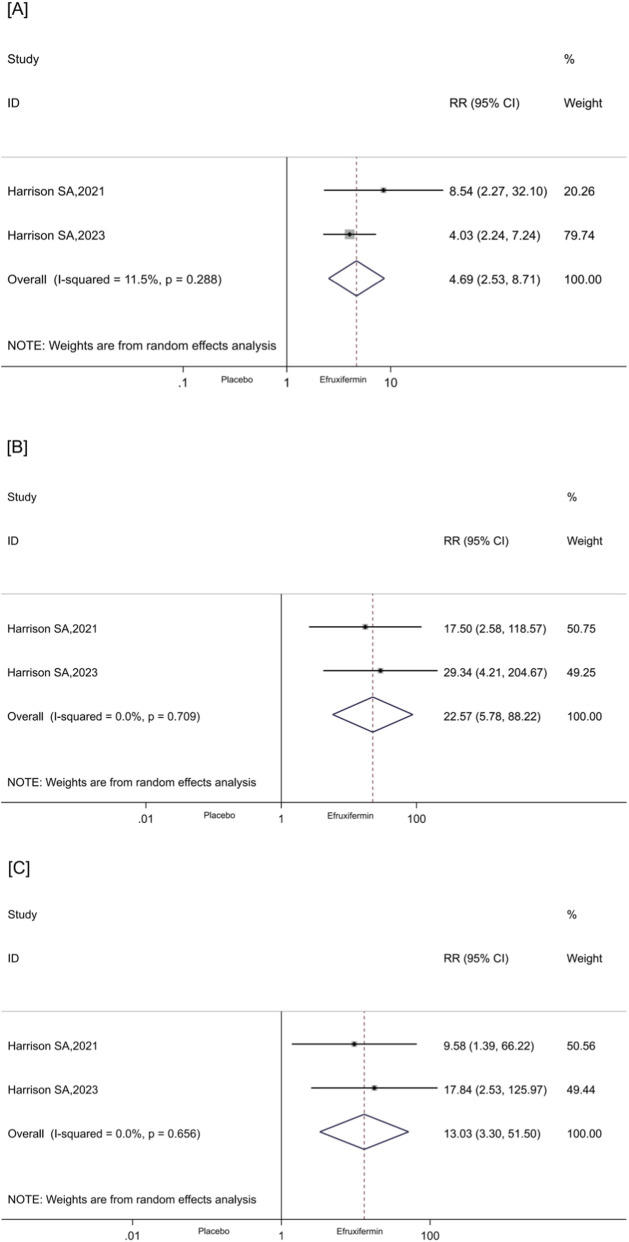
**(A–C)**. Forest plots for the meta-analysis comparing the effects of efruxifermin versus placebo on HFF: **(A)** reduction in HFF ≥30%; **(B)** reduction in HFF ≥50%; **(C)** normalised liver fat ≤5%. HFF, hepatic fat fraction; RR, relative risk; CI, confidence interval.

Two RCTs (Harrison et al., 2023; Harrison et al., 2021a) evaluated the number of patients with NASH or MASH who achieved a reduction of at least 30% and 50% in HFF as assessed by MRI - Proton Density Fat Fraction (MRI - PDFF). The findings indicated that, in comparison to the placebo group, efruxifermin significantly increased the proportion of patients achieving a ≥30% reduction in HFF (RR: 4.69, 95% CI [2.53, 8.71], P < 0.001, I^2^ = 11.5%, Figure 6A) and a ≥50% reduction in HFF (RR: 22.57, 95% CI [5.78, 88.22], P < 0.001, I^2^ = 0.0%, Figure 6B). Moreover, efruxifermin significantly increased the proportion of patients achieving liver fat normalization, defined as ≤5% (RR: 13.03, 95% CI [3.30, 51.50], P < 0.001, I^2^ = 0.0%, Figure 6C).

#### 3.4.4 Markers of liver function

Three trials (Noureddin et al., 2025; Harrison et al., 2021a; Harrison et al., 2022) comparing efruxifermin to placebo demonstrated a significant reduction in LS mean alanine aminotransferase (ALT) levels in the efruxifermin group (WMD: −11.98 U/L, 95% CI [−22.48, −1.49], P = 0.025, Supplementary Figure S2). However, a high degree of statistical heterogeneity was noted across the studies (I^2^ = 85%). Two trials (Noureddin et al., 2025; Harrison et al., 2022) reported the LS mean changes from baseline in aspartate aminotransferase (AST), gamma-glutamyl transferase (GGT), and alkaline phosphatase (ALP). Specifically, efruxifermin resulted in a modest reduction in LS mean AST levels compared to placebo (WMD: −6.32 U/L, 95% CI [−10.22, −2.42], P = 0.001, I^2^ = 0.0%, Supplementary Figure S3) and a significant reduction in LS mean GGT levels (WMD: −21.28 U/L, 95% CI [−36.27, −6.29], P = 0.005, I^2^ = 0.0%, Supplementary Figure S4). However, no significant difference in LS mean ALP levels was observed between the two groups (WMD: −0.67 U/L, 95% CI [−11.13, 12.46], P = 0.912, I^2^ = 54.1%, Supplementary Figure S5).

#### 3.4.5 Adverse events (AEs)

**FIGURE 7:**
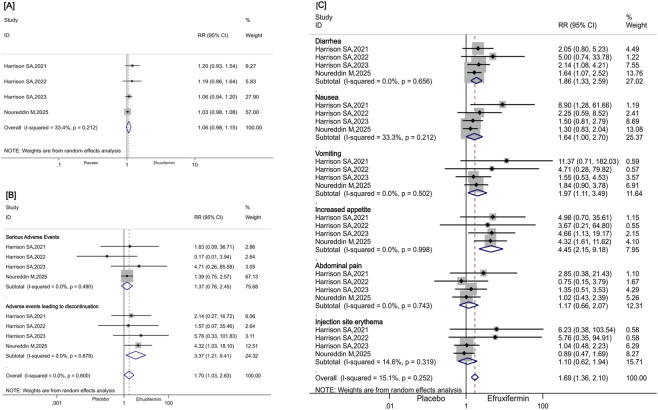
**(A–C)**. Forest plot for meta-analysis comparing the effects of efruxifermin with placebo on TEAEs, serious adverse events, adverse events leading to discontinuation, and adverse events during treatment that occurred in ≥15% of total participants. TEAEs, treatment-emergent adverse events, RR, risk ratios, CI, confidence interval.

Compared to placebo, efruxifermin did not significantly increase the incidence of TEAEs (RR: 1.06, 95% CI [0.98, 1.15], P = 0.163, I^2^ = 33.4%, Figure 7A), nor did it significantly elevate the incidence of serious AEs (RR: 1.37, 95% CI [0.76, 2.45], P = 0.29, I^2^ = 0.0%, Figure 7B). However, efruxifermin was associated with a significant increase in the risk of treatment discontinuation due to AEs (RR: 3.37, 95% CI [1.21, 9.41], P = 0.02, I^2^ = 0.0%, Figure 7B). Additionally, efruxifermin significantly increased the rate of diarrhea (RR: 1.86, 95% CI [1.33, 2.59], P < 0.001, I^2^ = 0.0%, Figure 7C), vomiting (RR: 1.97, 95% CI [1.11, 3.49], P = 0.020, I^2^ = 0.0%, Figure 7C),increased appetite (RR: 4.45, 95% CI [2.15, 9.18], P < 0.001, I^2^ = 0.0%, Figure 7C),while the incidence of abdominal pain (RR: 1.17, 95% CI [0.66, 2.07], P = 0.583, I^2^ = 0.0%, Figure 7C), nausea (RR: 1.64, 95% CI [1.00, 2.70], P = 0.051, I^2^ = 33.3%, Figure 7C), and injection site erythema (RR: 1.10, 95% CI [0.62, 1.94], P = 0.793, I^2^ = 14.6%, Figure 7C) was similar between efruxifermin and placebo.

We also observed that the incidence of fatigue (RR: 0.97, 95% CI [0.39, 2.44], P = 0.951, I^2^ = 56.7%), headache (RR: 1.21, 95% CI [0.71, 2.05], P = 0.488, I^2^ = 0.0%), injection site bruising (RR: 1.17, 95% CI [0.69, 1.97], P = 0.561, I^2^ = 0.0%), nasopharyngitis (RR: 1.31, 95% CI [0.76, 2.28], P = 0.329, I^2^ = 0.0%), sinusitis (RR: 0.88, 95% CI [0.50, 1.53], P = 0.639, I^2^ = 0.0%), and constipation (RR: 1.86, 95% CI [0.63, 5.48], P = 0.257, I^2^ = 0.0%) was similar between efruxifermin and placebo (Supplementary Figure S6).

#### 3.4.6 Sensitivity analysis

Regarding AEs leading to discontinuation and histological outcomes based on liver biopsy, after switching from a random-effects model to a fixed-effects model, the statistical significance of all results remained unchanged, and there were no significant changes in the level of heterogeneity (Supplementary Figures S7,S8). For the core outcome of fibrosis regression by ≥1 stage and no worsening in NASH/MASH, the RR consistently maintained statistical significance, irrespective of the study excluded. Furthermore, the RR value closely approximated the combined result of all studies, indicating a high stability of the effect size (Supplementary Figure S9–S12). For AEs leading to discontinuation, the difference became statistically insignificant (P = 0.206) after excluding the study by Noureddin et al. (Supplementary Figures S13–S16).

#### 3.4.7 GRADE assessment

The GRADE evidence profile was presented in Supplementary Table S3. The certainty of evidence was low for NASH/MASH resolution and improvement in fibrosis stage, decrease in NAS≥2 with no worsening in fibrosis stage, achieving a ≥2 stage improvement in fibrosis with no worsening of steatohepatitis, and absolute change in ALT. The remaining outcomes were of moderate certainty. Consequently, the current conclusions should be interpreted with caution.

## 4 Discussion

NAFLD/MASLD is a global health concern (Harrison et al., 2021b) that affects over 30% of the global population and is the most prevalent chronic liver disorder worldwide (Devarbhavi et al., 2023). Among patients diagnosed with NAFLD, approximately 10% progress to NASH (Dufour, 2024). The prevalence of NASH/MASH continues to rise in parallel with the increasing prevalence of obesity and metabolic comorbidities (Rinella et al., 2023a), the estimated global prevalence of NASH is approximately 4 to 6%, and the associated socioeconomic costs are high (Harrison et al., 2024b). Given the sharp rise in the global prevalence of MASH, there is an urgent need to develop new therapeutic strategies to address the increasing clinical demands.

In the treatment of NAFLD/MASLD, multiple therapeutic targets have been extensively investigated, including FGF, thyroid hormone receptor-β (THR-β), peroxisome proliferator-activated receptors (PPAR), glucagon-like peptides (GLP), and various drugs aimed at hepatic steatosis synthesis, inflammatory pathways, genetic components, and fibrosis mechanisms (Wei et al., 2024; Sangro et al., 2023). Among these numerous targets, FGF-21 stands out as a highly promising candidate for MASLD treatment, as it plays a crucial role in reducing lipogenesis and enhancing hepatic insulin sensitivity (Falamarzi et al., 2022). Previous preclinical studies have consistently demonstrated that FGF-21 not only offers significant therapeutic benefits for NAFLD/MASLD but also exhibits promising efficacy in related conditions such as obesity, insulin resistance, dyslipidemia, hyperglycemia, and atherosclerosis (Jin et al., 2023). Although a meta-analysis indicated that the first candidate drug, pegbelfermin (BMS-986036), could reduce aminotransferase levels in patients with NASH (Lu et al., 2024), its development was ultimately discontinued after a phase IIb study failed to meet its primary endpoint (Loomba et al., 2024). In contrast, efruxifermin and pegozafermin (Loomba et al., 2023), which are long-acting FGF-21 analogs with extended half-lives and optimized dosing intervals, are garnering widespread attention. Existing clinical trial data suggest that both compounds exhibit potential for treating MASLD.

This systematic review and meta-analysis comprehensively evaluated the clinical efficacy and safety of efruxifermin in the treatment of NASH/MASH. Data from four RCTs involving a total of 419 patients were pooled and analyzed. The results indicated that, compared to placebo, efruxifermin exhibited superior efficacy in achieving at least a 1-stage improvement in fibrosis without worsening NASH/MASH, as well as in the resolution of NASH/MASH and improvement in fibrosis stage. Furthermore, it achieved at least a 2-point improvement in NAS without worsening fibrosis. Resmetirom (Rezdiffra™), an oral THR-β agonist, gained FDA approval in March 2024 as the first drug for noncirrhotic MASH with moderate to advanced fibrosis (F2–F3) (Keam, 2024). At week 52 in the Phase 3 MAESTRO-NASH trial (Harrison et al., 2024b), resmetirom treatment resulted in ≥1-stage fibrosis improvement without NASH worsening in 24.2% (80 mg) and 25.9% (100 mg) of patients (placebo: 14.2%), and NASH resolution without fibrosis worsening in 25.9% (80 mg) and 29.9% (100 mg) of patients (placebo: 9.7%). A network meta-analysis (Souza et al., 2025) of 29 RCTs (N = 9,324) showed that for MASH resolution without fibrosis worsening, pegozafermin (RR: 8.65), survodutide (RR: 6.62), tirzepatide (RR: 4.65), resmetirom (RR: 2.54), and lanifibranor (RR: 1.93) all outperformed placebo, while for ≥1-stage fibrosis improvement without MASH worsening, pegozafermin (RR: 3.46), survodutide (RR: 1.86), tirzepatide (RR: 1.77), and resmetirom (RR: 1.64) were also significantly more effective than placebo. In Our meta-analysis, efruxifermin showed significant efficacy *versus* placebo, increasing rates of NASH/MASH resolution with fibrosis improvement (RR: 5.15) and ≥1-stage fibrosis improvement without NASH/MASH worsening (RR: 2.18). The efficacy estimates for efruxifermin derived from our analysis appear to be higher than those reported for the FDA-approved resmetirom; concurrently, they seem comparable to those of GLP-1 receptor agonists, the FGF21 analog pegozafermin, and PPAR agonists. However, this indirect comparison is significantly constrained by clinical heterogeneity (e.g., variations in baseline patient profiles and trial designs) and methodological differences across the analyses. Consequently, such cross-trial comparisons should be interpreted with caution. Definitive conclusions regarding their relative efficacy must await validation through head-to-head RCTs. In patients with compensated cirrhosis due to MASH, no significant improvement in liver fibrosis was observed with efruxifermin treatment at 36 weeks; by 96 weeks, the efruxifermin 50 mg group demonstrated potential benefits in liver fibrosis improvement. Given that fibrosis stage is the strongest predictor of adverse clinical outcomes in NASH/MASH, (Angulo et al., 2015; Sanyal, 2019), it is essential to conduct longer-term studies and include more diverse patient populations to comprehensively evaluate clinical outcomes, safety, and the generalizability of the findings, as well as to assess the benefits of long-term treatment. Furthermore, phase 3 trials are necessary to thoroughly evaluate the clinical efficacy of efruxifermin.

In addition, our meta-analysis demonstrated that efruxifermin could improve hepatic steatosis, non-invasive biomarkers associated with fibrosis, and liver function indices. In terms of liver function, efruxifermin significantly reduced ALT levels (WMD: −11.98 U/L), with concomitant improvements in AST (WMD: −6.32 U/L) and GGT (WMD: −21.28 U/L). Although the reduction in ALT observed was below the threshold of ≥17 U/L, which is associated with histological response (Loomba et al., 2019), it is important to note that the treatment duration in the included studies was relatively short, primarily spanning 16–24 weeks. With a longer treatment duration, there is potential for further improvement in ALT levels. Furthermore, the observed concordance in the reductions of ALT, AST, and GGT, alongside histological improvement and decreased hepatic fat, suggests a synchronous biochemical amelioration. This likely reflects underlying benefits in liver pathology, including the alleviation of steatosis and inflammation. This study found that efruxifermin was associated with common adverse reactions of injection site erythema and gastrointestinal symptoms (including diarrhea, vomiting, and increased appetite), none of which escalated to serious AEs. However, patients with NASH/MASH who received efruxifermin exhibited an increased risk of treatment discontinuation due to AEs, although this finding lacked robustness in sensitivity analyses. This observation underscores the necessity of monitoring the potential impact of the drug on treatment adherence, thereby emphasizing the importance of balancing efficacy and safety in clinical applications.

Although this meta-analysis showed good homogeneity for primary and most outcomes (I^2^ = 0%), high heterogeneity was observed for ALT (I^2^ = 85%) and fatigue (I^2^ = 56.7%). This heterogeneity may arise from several factors. First, patient populations were heterogeneous, encompassing fibrosis stages from F1-F3 to F4, with varying baseline levels of hepatic inflammation, hepatocellular injury, and metabolic profiles, potentially leading to differential treatment responses. Second, dosing regimens differed across trials, which pooled efruxifermin doses ranging from 28 mg to 70 mg. Third, treatment duration varied from 12 to 96 weeks, which may influence the effect size. Consequently, the pooled estimates for these highly heterogeneous outcomes should be interpreted with caution.

The sensitivity analysis revealed that the risk difference for treatment discontinuation due to adverse events lost statistical significance (P = 0.206) after the exclusion of the study by Noureddin et al. This phenomenon may be attributed to several factors: First, the study by Noureddin et al. had the largest sample size. Second, it exclusively enrolled patients with MASH-related compensated cirrhosis (F4 stage) and featured a significantly longer follow-up period of 96 weeks. Patients with F4 cirrhosis, due to their more advanced underlying liver disease, may be more susceptible to treatment discontinuation resulting from adverse events. Furthermore, the extended exposure time in this trial could have resulted in cumulative risks that are not evident in shorter-term studies. Overall, this safety conclusion lacks robustness and should be interpreted with caution.

In June 2025, Zhong et al. (Zhong et al., 2025) published a network meta-analysis on the pharmacological treatment of MASLD, aiming to systematically compare the efficacy and safety of various drugs in reducing hepatic steatosis and fibrosis over a 24-week period. In contrast to that study, our meta-analysis does not impose specific restrictions on the duration of drug intervention. Unlike the meta-analyses conducted by Souza et al. (Souza et al., 2025) and Jeong et al. (Jeong et al., 2024), this study specifically focuses on efruxifermin. Furthermore, in comparison with the systematic review and meta-analysis by Li et al. (Li et al., 2025), which assessed the efficacy of efruxifermin in improving liver fibrosis in patients with NASH/MASH, this study incorporates the latest data from the phase 2b clinical trial of efruxifermin conducted in patients with compensated cirrhosis (stage F4) due to MASH, and comprehensively includes important outcome measures such as hepatic steatosis.

This meta-analysis exhibits several significant strengths. To the best of our knowledge, this study is the first to incorporate data from the Noureddin et al. clinical trial of efruxifermin into a systematic review and meta-analysis. Furthermore, this meta-analysis not only encompasses histological outcomes but also includes data on fibrosis-related non-invasive biomarkers, hepatic steatosis, liver function markers, and adverse events. The findings of this study provide robust evidence supporting the conduct of larger-scale clinical trials of efruxifermin and its potential clinical applications in the treatment of MASH and MASH-related liver fibrosis.

## 5 Study limitations

This study acknowledges several limitations. First, the short follow-up duration and limited sample size represent the primary limitations of this research. Second, owing to the limited number of included studies, formal assessment of publication bias using funnel plots or Egger’s test was not feasible. Consequently, the potential impact of publication bias on the pooled effect size cannot be ruled out. Additionally, due to the limited number of included RCTs and the constraints of the original data, our meta-analysis was unable to conduct meaningful, statistically powered subgroup analyses to explore the potential differential effects of efruxifermin based on dosage (28 mg, 50 mg, 70 mg) or baseline fibrosis stage (e.g., F1-F3 *versus* F4 cirrhosis). The absence of these subgroup analyses regarding dosage and fibrosis staging limits the clinical guidance necessary for individualized medication. Meanwhile, we were unable to conduct subgroup analyses to identify the relevant influencing factors contributing to the high heterogeneity observed in certain outcomes, such as ALT. This limitation undermines the robustness of the associated findings. Furthermore, the baseline characteristics of the study populations (including gender, race, age, and baseline blood glucose levels) may have influenced the study results. Unfortunately, limitations in the original data precluded further subgroup analysis to explore their potential effects. Consequently, the results of this meta-analysis should be interpreted with caution.

## 6 Conclusion

Efruxifermin may represent a promising therapeutic option for NASH/MASH. Given the limitations in both the number and short follow-up duration of the included RCTs, the conclusions should be interpreted with caution. Further large-scale, multicenter, long-term, and high-quality RCTs are necessary to validate these results in diverse populations.

## Data Availability

The original contributions presented in the study are included in the article/Supplementary Material, further inquiries can be directed to the corresponding authors.
