# Supplementary material for: Efficacy and safety of efruxifermin for patients with NASH/MASH: an updated systematic review and meta-analysis
Source: Front Pharmacol. 2026 Apr 2;16:1731065. doi: 10.3389/fphar.2025.1731065 (PMC13084562; doi:10.3389/fphar.2025.1731065)
Supplement: Supplementary file 1 [file DataSheet1.pdf]

Supplementary Appendix

CONTENTS

Table S1. Database search strategy (PubMed).

Table S2. Study details and participant baseline characteristics included in the meta-analysis.

Figure S1. Forest plots of achieving a ≥2 stage improvement in fibrosis with no worsening of steatohepatitis in the efruxifermin and placebo groups.

Figure S2. Forest plot of the meta-analysis comparing the effects of efruxifermin versus placebo on the least squares (LS) mean change in ALT (U/L).

Figure S3. Forest plot of the meta-analysis comparing the effects of efruxifermin versus placebo on the LS mean change in AST (U/L).

Figure S4. Forest plot of the meta-analysis comparing the effects of efruxifermin versus placebo on the LS mean change in GGT (U/L).

Figure S5. Forest plot of the meta-analysis comparing the effects of efruxifermin versus placebo on the LS mean change in ALP (U/L).

Figure S6. Forest plot for meta-analysis comparing the effects of efruxifermin with placebo on other interested adverse events.

Figure S7. Sensitivity Analysis: Forest plot for meta-analysis of efruxifermin versus placebo on Histological assessment of liver biopsy.

Figure S8. Sensitivity Analysis: Forest plot for meta-analysis comparing efruxifermin with placebo on adverse events leading to discontinuation.

Figure S9-12. Sensitivity analysis: Forest plot for meta-analysis comparing the effects of efruxifermin with placebo on fibrosis regression by at least one stage and no worsening in NASH/MASH.

Figure S13-16. Sensitivity analysis: Forest plot for meta-analysis comparing efruxifermin versus placebo on adverse events leading to treatment discontinuation.

Table S3. GRADE evidence profile.

PRISMA 2020 Checklist

Section and Topic	Item #	Checklist item	Location where item is reported
**TITLE**			
Title	1	Identify the report as a systematic review.	Title
**ABSTRACT**			
Abstract	2	See the PRISMA 2020 for Abstracts checklist.	Abstract
**INTRODUCTION**			
Rationale	3	Describe the rationale for the review in the context of existing knowledge.	Introduction
Objectives	4	Provide an explicit statement of the objective(s) or question(s) the review addresses.	Introduction
**METHODS**			
Eligibility criteria	5	Specify the inclusion and exclusion criteria for the review and how studies were grouped for the syntheses.	2.2 Study selection
Information sources	6	Specify all databases, registers, websites, organisations, reference lists and other sources searched or consulted to identify studies. Specify the date when each source was last searched or consulted.	2.1 Data sources and searches
Search strategy	7	Present the full search strategies for all databases, registers and websites, including any filters and limits used.	2.1 Data sources and searches, and Supplementary Tables S1 (not all databases)
Selection process	8	Specify the methods used to decide whether a study met the inclusion criteria of the review, including how many reviewers screened each record and each report retrieved, whether they worked independently, and if applicable, details of automation tools used in the process.	2.2 Study selection
Data collection process	9	Specify the methods used to collect data from reports, including how many reviewers collected data from each report, whether they worked independently, any processes for obtaining or confirming data from study investigators, and if applicable, details of automation tools used in the process.	2.3 Data extraction
Data items	10a	List and define all outcomes for which data were sought. Specify whether all results that were compatible with each outcome domain in each study were sought (e.g. for all measures, time points, analyses), and if not, the methods used to decide which results to collect.	2.2 Study selection,Tables 1 and Tables S2
	10b	List and define all other variables for which data were sought (e.g. participant and intervention characteristics, funding sources). Describe any assumptions made about any missing or unclear information.	2.3 Data extraction, Tables 1 and Tables S2
Study risk of bias assessment	11	Specify the methods used to assess risk of bias in the included studies, including details of the tool(s) used, how many reviewers assessed each study and whether they worked independently, and if applicable, details of automation tools used in the process.	2.4 Quality assessment
Effect measures	12	Specify for each outcome the effect measure(s) (e.g. risk ratio, mean difference) used in the synthesis or presentation of results.	2.5 Data synthesis and analysis
Synthesis methods	13a	Describe the processes used to decide which studies were eligible for each synthesis (e.g. tabulating the study intervention characteristics and comparing against the planned groups for each synthesis (item #5)).	Tables 1 and Tables S2
	13b	Describe any methods required to prepare the data for presentation or synthesis, such as handling of missing summary statistics, or data	2.5 Data

PRISMA 2020 Checklist

Section and Topic	Item #	Checklist item	Location where item is reported
		conversions.	synthesis and analysis
	13c	Describe any methods used to tabulate or visually display results of individual studies and syntheses.	2.5 Data synthesis and analysis
	13d	Describe any methods used to synthesize results and provide a rationale for the choice(s). If meta-analysis was performed, describe the model(s), method(s) to identify the presence and extent of statistical heterogeneity, and software package(s) used.	2.5 Data synthesis and analysis
	13e	Describe any methods used to explore possible causes of heterogeneity among study results (e.g. subgroup analysis, meta-regression).	Not involved
	13f	Describe any sensitivity analyses conducted to assess robustness of the synthesized results.	2.5 Data synthesis and analysis
Reporting bias assessment	14	Describe any methods used to assess risk of bias due to missing results in a synthesis (arising from reporting biases).	Not involved
Certainty assessment	15	Describe any methods used to assess certainty (or confidence) in the body of evidence for an outcome.	2.4 Quality assessment
**RESULTS**			
Study selection	16a	Describe the results of the search and selection process, from the number of records identified in the search to the number of studies included in the review, ideally using a flow diagram.	3.1 Study selection and Figure 1
	16b	Cite studies that might appear to meet the inclusion criteria, but which were excluded, and explain why they were excluded.	Figure 1
Study characteristics	17	Cite each included study and present its characteristics.	3.2 Study characteristics, Table 1,and Supplemental Table S2
Risk of bias in studies	18	Present assessments of risk of bias for each included study.	3.3 Risk of bias, and Figure 2-3
Results of individual studies	19	For all outcomes, present, for each study: (a) summary statistics for each group (where appropriate) and (b) an effect estimate and its precision (e.g. confidence/credible interval), ideally using structured tables or plots.	3.4 Results and Supplemental Table S2
Results of syntheses	20a	For each synthesis, briefly summarise the characteristics and risk of bias among contributing studies.	3.4 Results
	20b	Present results of all statistical syntheses conducted. If meta-analysis was done, present for each the summary estimate and its precision (e.g. confidence/credible interval) and measures of statistical heterogeneity. If comparing groups, describe the direction of the effect.	3.4 Results, Figure 4-7, and Supplementary eFigure 1-6
	20c	Present results of all investigations of possible causes of heterogeneity among study results.	4 Discussion
	20d	Present results of all sensitivity analyses conducted to assess the robustness of the synthesized results.	3.4.6 Sensitivity analysis and Supplementary

PRISMA 2020 Checklist

Section and Topic	Item #	Checklist item	Location where item is reported
			eFigure 7-16
Reporting biases	21	Present assessments of risk of bias due to missing results (arising from reporting biases) for each synthesis assessed.	Not involved
Certainty of evidence	22	Present assessments of certainty (or confidence) in the body of evidence for each outcome assessed.	3.4.7 GRADE assessment, Supplemental Table S3
**DISCUSSION**			
Discussion	23a	Provide a general interpretation of the results in the context of other evidence.	4 Discussion
	23b	Discuss any limitations of the evidence included in the review.	4 Discussion
	23c	Discuss any limitations of the review processes used.	4 Discussion
	23d	Discuss implications of the results for practice, policy, and future research.	4 Discussion
**OTHER INFORMATION**			
Registration and protocol	24a	Provide registration information for the review, including register name and registration number, or state that the review was not registered.	2 Methods
	24b	Indicate where the review protocol can be accessed, or state that a protocol was not prepared.	2 Methods
	24c	Describe and explain any amendments to information provided at registration or in the protocol.	Not involved
Support	25	Describe sources of financial or non-financial support for the review, and the role of the funders or sponsors in the review.	Funding
Competing interests	26	Declare any competing interests of review authors.	Conflict of interest
Availability of data, code and other materials	27	Report which of the following are publicly available and where they can be found: template data collection forms; data extracted from included studies; data used for all analyses; analytic code; any other materials used in the review.	Data availability statement

Table S1 Database search strategy (PubMed).

#1	"Non-alcoholic Fatty Liver Disease"[Mesh]
#2	((((((((((((Non alcoholic Fatty Liver Disease[Title/Abstract]) OR (NAFLD[Title/Abstract])) OR (Nonalcoholic Fatty Liver Disease[Title/Abstract])) OR (Fatty Liver, Nonalcoholic[Title/Abstract])) OR (Fatty Livers, Nonalcoholic[Title/Abstract])) OR (Liver, Nonalcoholic Fatty[Title/Abstract])) OR (Livers, Nonalcoholic Fatty[Title/Abstract])) OR (Nonalcoholic Fatty Live[Title/Abstract])) OR (Nonalcoholic Fatty Livers[Title/Abstract])) OR (Nonalcoholic Steatohepatitis[Title/Abstract])) OR (Nonalcoholic Steatohepatitides[Title/Abstract])) OR (Steatohepatitides, Nonalcoholic[Title/Abstract])) OR (Steatohepatitis, Nonalcoholic[Title/Abstract])) OR (Nash[Title/Abstract])
#3	#1 OR #2
#4	((Efruxifermin[Title/Abstract]) OR (EFX[Title/Abstract])) OR (AKR-001[Title/Abstract])
#5	#3 AND #4

Table S2. Study details and participant baseline characteristics included in the meta-analysis.

Study	Harrison SA,2021	Harrison SA,2022	Harrison SA,2023	Noureddin M,2025
Research type	RCT	RCT	RCT	RCT
Phase	Phase 2a	Phase 2a	Phase 2b	Phase 2b
NCT ID	NCT03976401	NCT03976401	NCT04767529	NCT05039450
Population	Biopsy-proven NASH, F1 - F3	NASH, F4	NASH, F2 or F3	MASH, F4
Major inclusion criteria	BMI > 25 kg/m^2; confirmation of ≥ 10% liver fat content on MRI-PDFF at screening; have had a liver biopsy within 180 days of randomization with fibrosis stage 1 to 3 and a NAS of ≥ 4 with at least a score of 1 in each of the following NAS components: Steatosis (scored 0 to 3), Ballooning degeneration (scored 0 to 2), and Lobular inflammation (scored 0 to 3)	Documented compensated cirrhosis (F4) due to NASH within 24 months of screening with no evidence of competing etiology, and at least 1 co-existing or historic metabolic comorbidity	Biopsy-proven NASH. Must have had a liver biopsy obtained ≤ 180 days prior to randomization with F2–F3 and a NAS of ≥ 4 with at least a score of 1 in each of the following NAS components: Steatosis (scored 0–3); Ballooning degeneration (scored 0–2); Lobular inflammation (scored 0–3); History or presence of 2 of 4 components of metabolic syndrome (obesity, dyslipidaemia, elevated blood pressure,elevated fasting glucose) or T2D	Had liver histologic features consistent with MASH, and had compensated cirrhosis, which was defined as stage 4 fibrosis with a Child–Pugh score of 5 or 6 (class A, the mildest stage of chronic liver disease) on a scale ranging from 5 to 15. The patients also had type 2 diabetes or two components of metabolic syndrome (obesity, dyslipidemia, elevated blood pressure, and elevated fasting glucose level)
Country	United States, Puerto Rico	United states	United states	United States, Puerto Rico, and Mexico
Treatment duration (weeks)	16	16	24	96
Sponsor	Akero Therapeutics	Akero Therapeutics	Akero Therapeutics	Akero Therapeutics
Group	N = 80;<br>placebo (n = 21);<br>Efruxifermin (n = 59);<br>Efruxifermin 28mg (n = 19);	N = 30;<br>placebo (n = 10);<br>Efruxifermin 50mg(n=20)	N = 128;<br>placebo (n=43);<br>Efruxifermin (n = 85);<br>Efruxifermin 28mg (n = 42);	N = 181;<br>placebo (n = 61);<br>Efruxifermin (120);<br>Efruxifermin 28mg (n = 57);

		Efruxifermin 50mg (n = 20);<br>Efruxifermin 70 mg (n = 20)		Efruxifermin 50mg (n = 43)	Efruxifermin 50mg (n = 63)
Interventions		Efruxifermin (28-50-70mg), placebo	Efruxifermin (50mg), placebo	Efruxifermin (28 - 50mg), placebo	Efruxifermin (28 - 50mg), placebo
Primary end point		Absolute reduction in HFF at week 12	Safety and tolerability of efruxifermin	Improvement in liver fibrosis by 1 or more stages without worsening of NASH	A reduction in fibrosis without a worsening of MASH on the basis of liver histologic testing at week 36
Secondary end points		LS mean relative change in HFF from baseline to week 12; the proportion of patients in the FAS with ⩾30% relative reduction of HFF at week 12; the change from baseline in levels of ALT at week 12	Change from baseline to week 16 in liver stiffness and non-invasive biomarkers of fibrosis: ELF score and serum levels of Pro-C3	Proportion of patients with NASH resolution, defined as score of 0 for ballooning, 0 or 1 for inflammation, any value for steatosis without worsening of liver fibrosis in liver fibrosis, and so on	A reduction in fibrosis without a worsening of MASH at week 96 and MASH resolution at weeks 36 and 96, and so on
Exploratory endpoints		Changes from baseline in body weight, waist-to-hip ratio at weeks 12, 16, and 20, and bone mineral density of the lumbar spine, femoral neck, and total hip (measured by DXA) at week 16, and so on	Changes in liver histopathology, as well as in markers of liver injury and of glucose and lipid metabolism	The proportion of patients with resolution of NASH and improvement in fibrosis, improvement in fibrosis by at least 2 stages without worsening of NASH, change from baseline in non-invasive markers of liver injury and normalisation of HFF	The change from baseline in noninvasive markers of fibrosis,along with lipoprotein levels, markers of glycemic control, body weight, and markers of liver injury
Target dose of intervention (mg)		28 - 70;once-weekly	50; once-weekly	28 - 50; once-weekly	28 - 50; once-weekly
Age, mean (SD), y		18 - 80years, 52.1 (12.2)	18 - 80years, 59.8 (11.6)	18 - 75years, 54.7 (10.4)	18 - 75years, 60.7 (8.2)
Male, n (%)		34 (43)	11 (37)	49 (38)	60 (33)
Race or ethnicity, n (%)					
	White	75 (94)	28 (93)	118 (92)	165 (91)
	Black or African American	4 (5)	1 (3)	4 (3)	NR
	Asian	1 (1)	0	5 (4)	NR

	Other	0	1 (3)	1 (1)	NR
	Hispanic or Latino	40 (50)	13 (43)	52 (41)	60 (33)
BMI, mean (SD), kg/m^2		37.6 (6.7)	37.0 (6.6)	38·0 (7·0)	35.8 (6.6)
Body weight,mean (SD), kg		103.5 (21.9)	105.0 (25.5)	104.8 (23.2)	98.2 (21.1)
Liver Histology					
	NAS, mean (SD)	5.3 (1.0)	3.8 (1.8)	5·4 (1·1)	3.9 (1.6)
	NAS ≥5, n (%)	63 (79)	NR	NR	NR
	NAS ≤3, n (%)	NR	NR	NR	66 (37)
	NAS >3, n (%)	NR	NR	NR	115 (64)
	Patients with F1, n (%)	29 (36)	0	0	0
	Patients with F2, n (%)	26 (33)	0	44 (34)	0
	Patients with F3, n (%)	25 (31)	0	84 (66)	0
MASH		80 (100)	30 (100)	128 (100)	142 (78)
Cryptogenic cirrhosi		0	0	0	39 (22)
Non-invasive measures					
	HFF by MRI–PDFF, mean (SD)	19.6 (6.9)	NR	17.7 (6.5)	NR
	HFF<15%, n (%)	24 (30)	NR	NR	NR
	HFF≥15%, n (%)	56 (70)	NR	NR	NR
Markers of fibrosis, mean (SD)					
	ELF test score	9.5 (0.8)	10.2 (1.1)	9.8 (0.8)	10.5 (0.8)
	Pro-C3, μg/L	17.1(7.4)	24.6 (23.3)	16.7 (6.7)	140.1 (68.7)
	LSM, kPa	NR	23.2 (11.6)	14.8 (6.2)	24.4 (13.3)
Markers of liver health, mean (SD)					

	ALT, U/L	55.7 (31.2)	32.0 (17.6)	58.5 (34.3)	39.6 (21.8)
	AST, U/L	39.9 (20.0)	30.6 (16.2)	50.5 (33.4)	36.7 (18.1)
	GGT, U/L	78.1 (96.4)	66.1 (36.0)	NR	95.6 (107.0)
	Urate, mg/dL	5.8 (1.3)	6.0 (1.2)	5.6 (1.4)	5.6 (1.5)
	ALP, U/L	NR	74.3 (23.1)	NR	80.1 (29.3)
Markers of liver function, mean (SD)					
	Bilirubin, mg/dl	NR	0.7 (0.3)	NR	0.7 (0.3)
	Albumin, g/dl	NR	4.2 (0.3)	NR	4.3 (0.3)
	PLT, 10^9/L	NR	183.4 (34.5)	NR	183.1 (65.7)
	Fibrinogen, mg/dl	NR	437.1 (77.0)	NR	NR
	International normalized ratio	NR	1.1 (0.1)	NR	1.1 (0.1)
	PAI-1, IU/ml	NR	14.3 (10.8)	NR	NR
	Model for end-stage liver disease score	NR	7.2 (1.0)	NR	7.8 (1.6)
	Child-Pugh score	NR	5.1 (0.2)	NR	5.0 (0.2)
	Child–Pugh score of 5, n	NR	NR	NR	177 (98)
Markers of lipid metabolism, mean (SD)					
	Triglycerides, mg/dl	185.7 (99.7)	130.3 (61.0)	160.7 (70.0)	150.5 (70.3)
	Total cholesterol, mg/dl	188.0 (43.7)	164.0 (40.8)	NR	NR
	HDL cholesterol, mg/dl	42.3 (10.3)	48.0 (13.6)	41.5 (8.8)	49.3 (14.5)
	Non-HDL-cholesterol, mg/dl	145.8 (43.4)	116.0 (41.2)	NR	119.7 (39.9)
	LDL cholesterol, mg/dl	108.7 (36.0)	89.9 (33.9)	100.4 (33.7)	89.3 (33.5)
	Apolipoprotein B, mg/dl	100.6 (28.2)	82.7 (25.9)	NR	NR
	Apolipoprotein C-III, mg/dl	10.4 (5.4)	7.3 (3.8)	NR	NR

	Lipoprotein-a, nmol/L	NR	57.5 (67.5)	NR	NR
	Beta -hydroxybutyrate, mmol/L	NR	0.1 (0.1)	NR	NR
	Bile acids, lmol/L	NR	9.8 (8.1)	NR	NR
Glycemic status					
	T2D, n (%)	41 (51)	15 (50)	90 (70)	145 (80)
	HbA1c (%), mean (SD)	6.3 (1.1)	6.3 (1.1)	6.8 (1.1)	6.7 (1.1)
	FPG (mg/dl), mean (SD)	127.2 (49.4)	112.5 (21.2)	NR	NR
	HOMA-IR	14.0 (15.8)	9.8 (5.8)	NR	11.7 (10.8)
	C-peptide (lg/L), mean (SD)	NR	5.3 (1.5)	NR	NR
	Insulin (mIU/L), mean (SD)	NR	32.5 (15.0)	NR	NR
	Adiponectin (mg/L), mean (SD)	4.5 (2.6)	5.5 (2.8)	3.5 (1.7)	5.1 (3.3)
Medication uses, n (%)					
	Metformin	30 (38)	NR	NR	NR
	Proton pump inhibitors	30 (38)	NR	NR	NR
	Statin	29 (37)	9 (30)	57 (45)	85 (47)
	Antidepressants or anxiolytics	44 (55)	NR	NR	NR
	GLP-1 analogues/GLP-1 RAs	NR	6 (20)	16 (13)	48 (27)
	SGLT2 inhibitors	NR	NR	19 (15)	NR
	Antidiabetic medication use	NR	NR	88 (70)	NR
Hypertension, n (%)		NR	NR	NR	146 (80)
Osteopenia, n (%)		NR	NR	NR	78 (43)

Abbreviations: ALP: Alkaline Phosphatase; ALT: Alanine Aminotransferase; AST: Aspartate Aminotransferase; BMI: Body Mass Index; ELF: Enhanced Liver Fibrosis; FPG: Fasting Plasma Glucose; GLP-1 RAs: Glucagon-like Peptide-1 Receptor Agonists; GGT: Gamma-Glutamyl

Transferase; HbA1c: Glycated Hemoglobin; HDL: High-Density Lipoprotein; HFF: Hepatic Fat Fraction; HOMA-IR: Homeostatic Model Assessment of Insulin Resistance; LDL: Low-Density Lipoprotein; LSM: Liver Stiffness Measurement; MASH: Metabolic Dysfunction-Associated Steatohepatitis; MRI-PDFF: MRI-Proton Density Fat Fraction; NASH: Non-Alcoholic Steatohepatitis; NAS: Non-Alcoholic Fatty Liver Disease Activity Score; PAI-1: Plasminogen Activator Inhibitor-1; PLT: Platelet; ProC3: Serum N-Terminal Type-III Collagen Pro-Peptide; SGLT2: Sodium-Glucose Co-Transporter 2; T2D: Type 2 Diabetes.

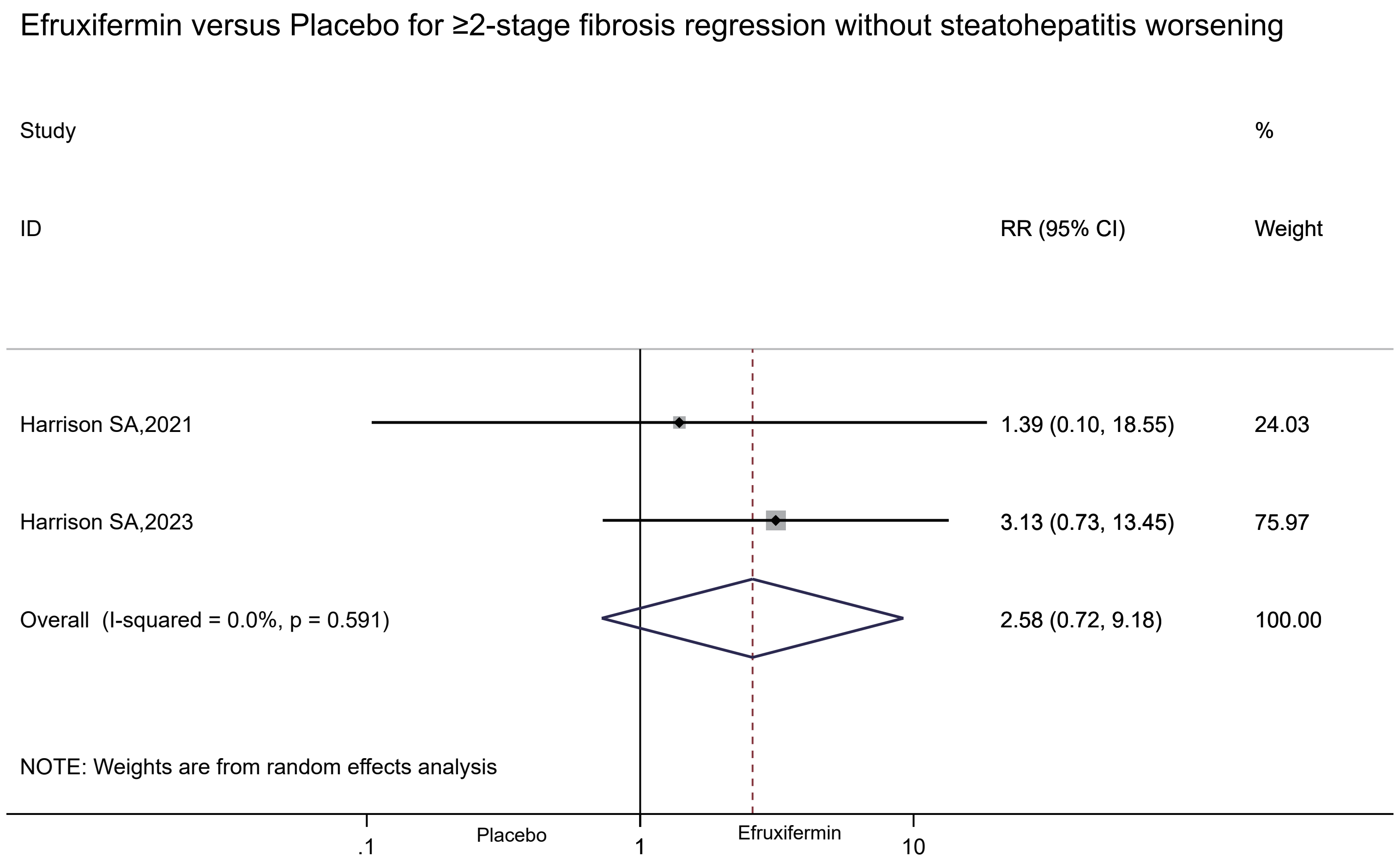

Figure S1. Forest plot for meta-analysis comparing the effects of efruxifermin with placebo on achieving a ≥2 stage improvement in fibrosis with no worsening of steatohepatitis. RR, risk ratios, CI, confidence interval.

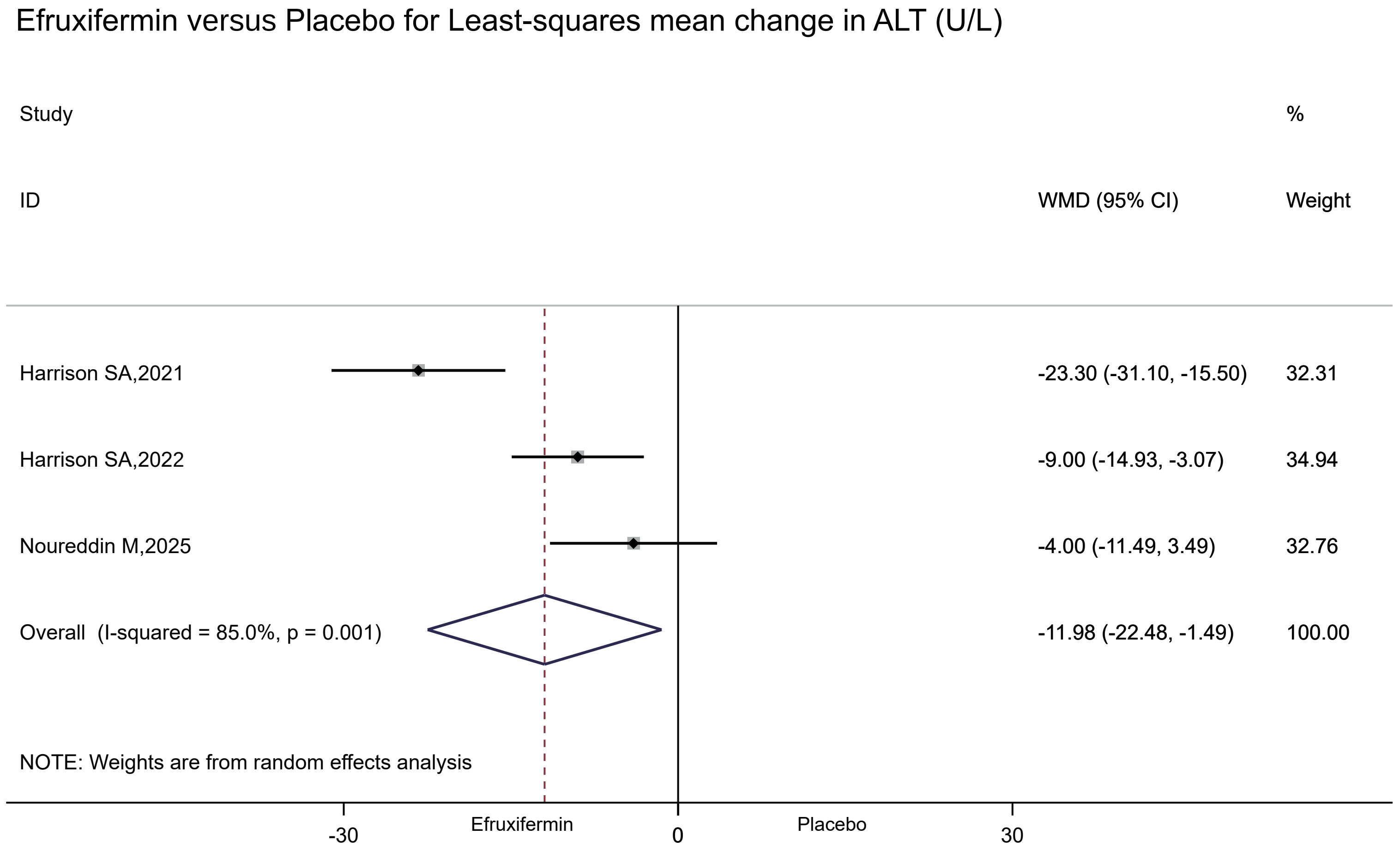

Figure S2. Forest plot of the meta-analysis comparing the effects of efruxifermin versus placebo on the least squares mean change in ALT (U/L). ALT, alanine aminotransferase; WMD, weighted mean difference; CI, confidence interval.

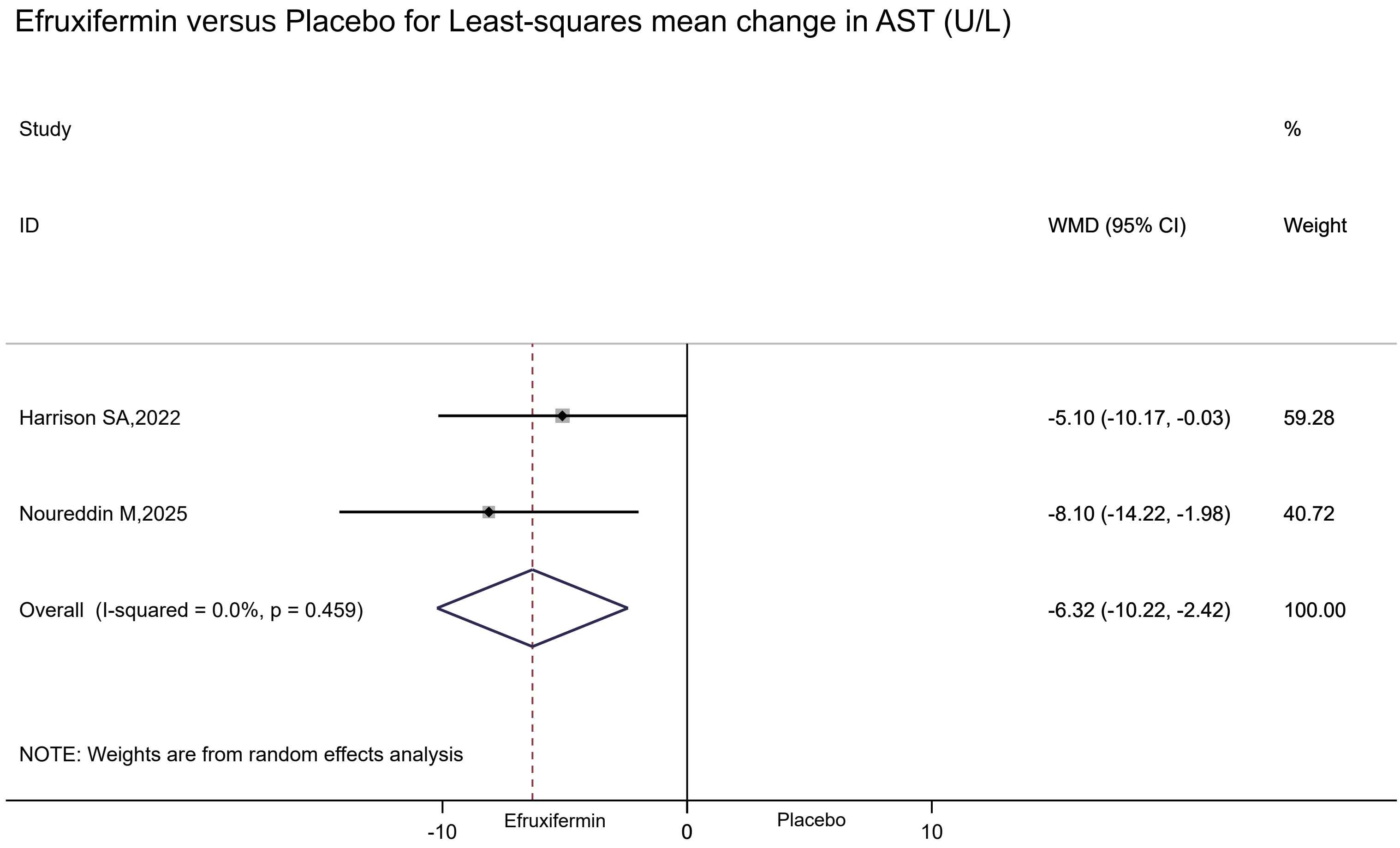

Figure S3. Forest plot of the meta-analysis comparing the effects of efruxifermin versus placebo on the least squares mean change in AST (U/L). AST, aspartate aminotransferase; WMD, weighted mean difference; CI, confidence interval.

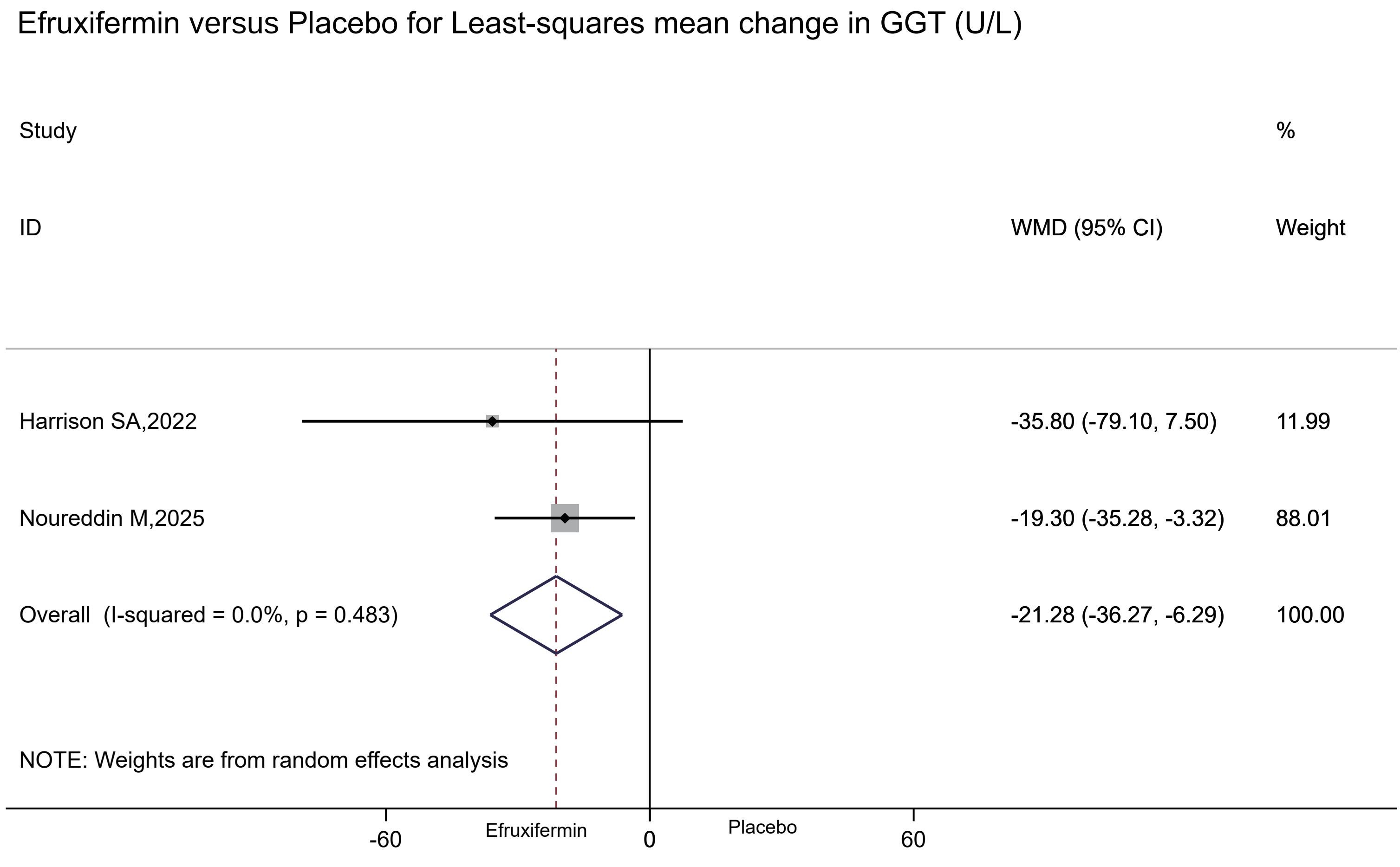

Figure S4. Forest plot of the meta-analysis comparing the effects of efruxifermin versus placebo on the least squares mean change in GGT (U/L). GGT, gamma-glutamyl transferase; WMD, weighted mean difference; CI, confidence interval.

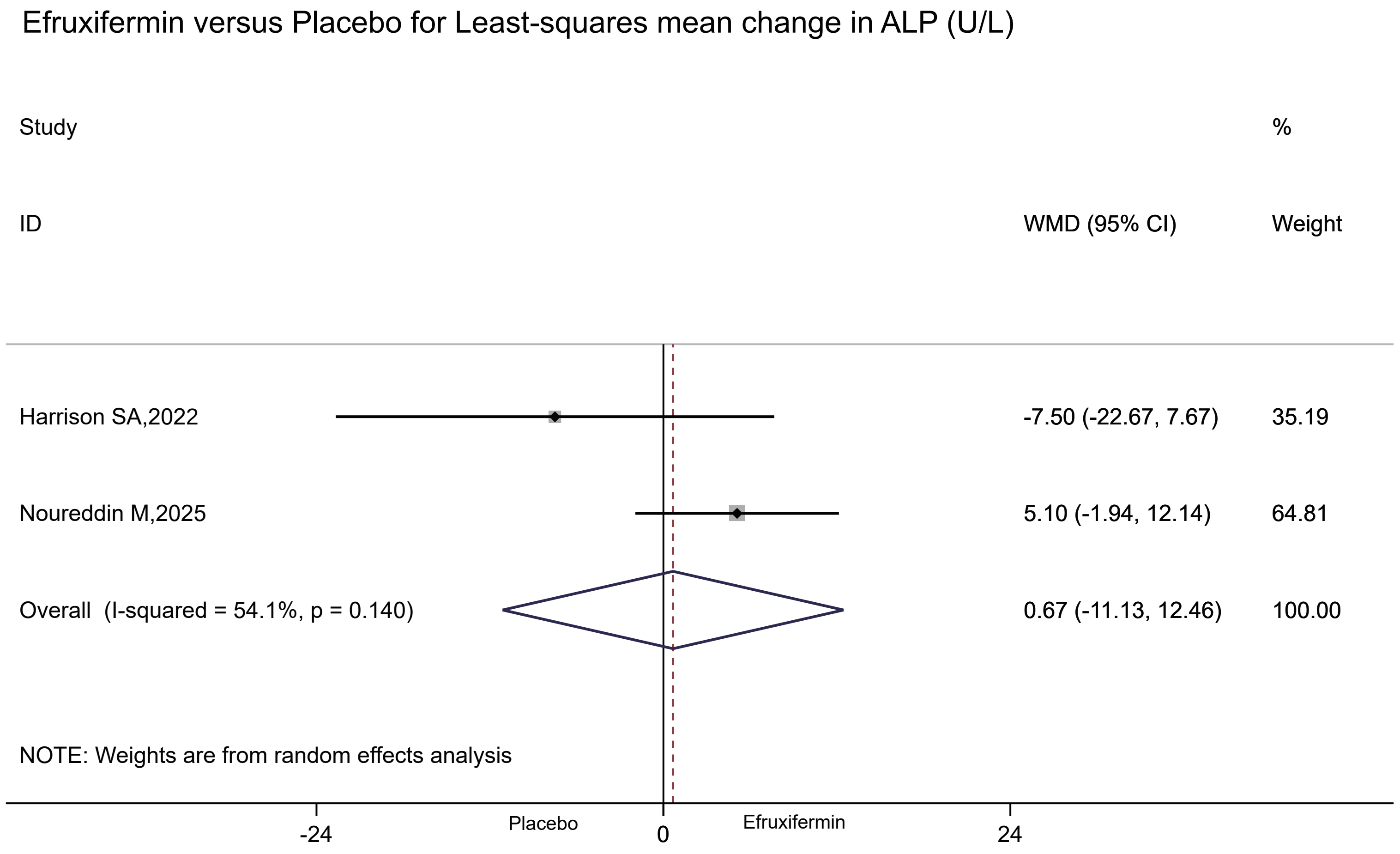

Figure S5. Forest plot of the meta-analysis comparing the effects of efruxifermin versus placebo on the least squares mean change in ALP (U/L). ALP, alkaline phosphatase; WMD, weighted mean difference; CI, confidence interval.

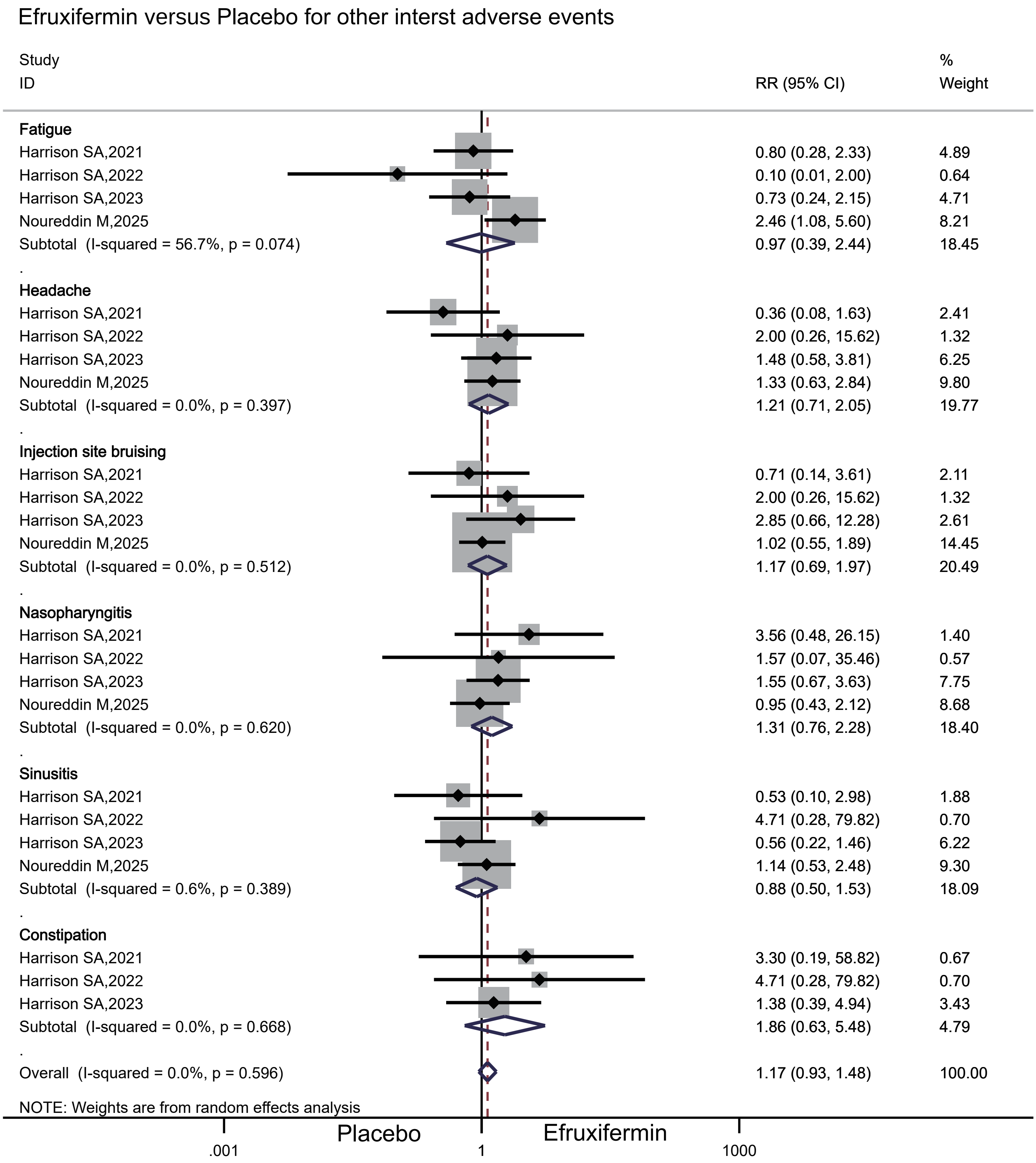

Figure S6. Forest plot for meta-analysis comparing the effects of efruxifermin with placebo on other interested adverse events. RR, risk ratios, CI, confidence interval.

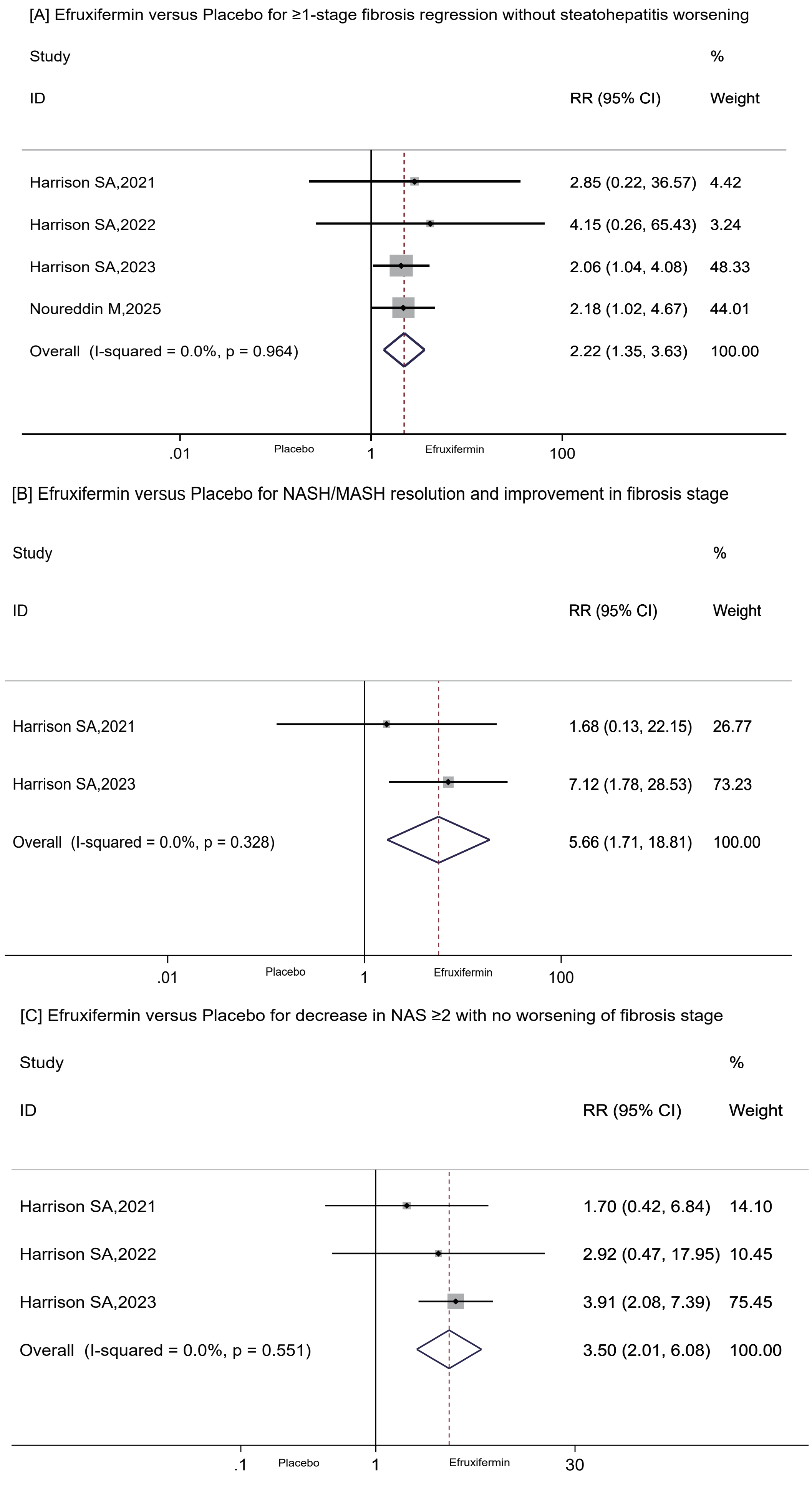

Figure S7A-C. Sensitivity Analysis: Forest plot for meta-analysis of efruxifermin versus placebo. Outcomes include fibrosis regression by ≥1 stage and no worsening in steatohepatitis, achieving NASH/MASH resolution and improvement in fibrosis stage, decrease in NAS by ≥2 without worsening the fibrosis stage. Results are presented after switching from a random-effect model to a fixed-effect model. NASH, Non-alcoholic steatohepatitis; MASH, Metabolic dysfunction-associated steatohepatitis; NAS, Non-alcoholic fatty liver disease activity score; RR, relative risk; CI, confidence interval.

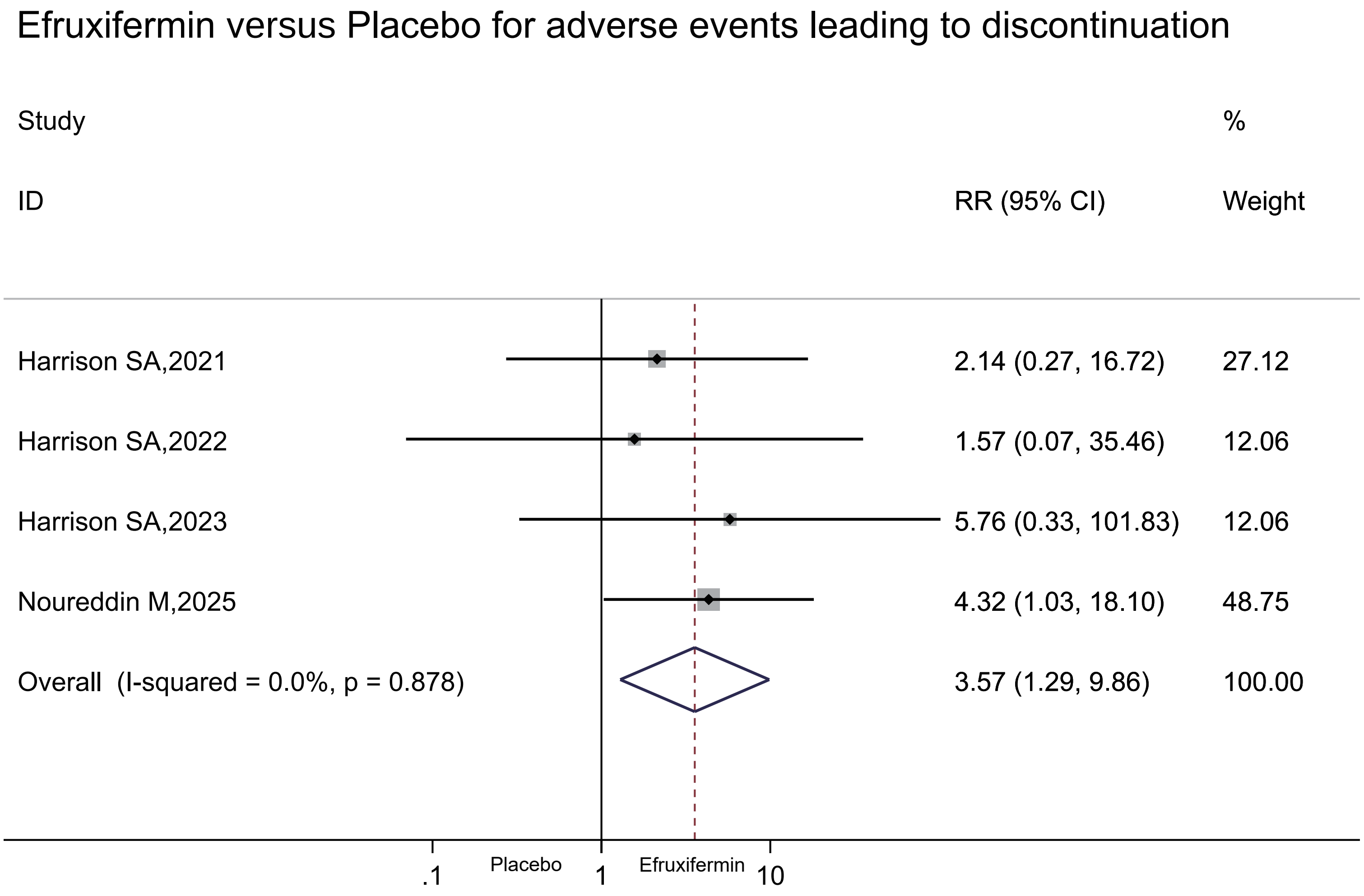

Figure S8. Sensitivity Analysis: Forest plot for meta-analysis comparing efruxifermin with placebo on adverse events leading to discontinuation, after switching from a random-effects model to a fixed-effects model. RR, relative risk; CI, confidence interval.

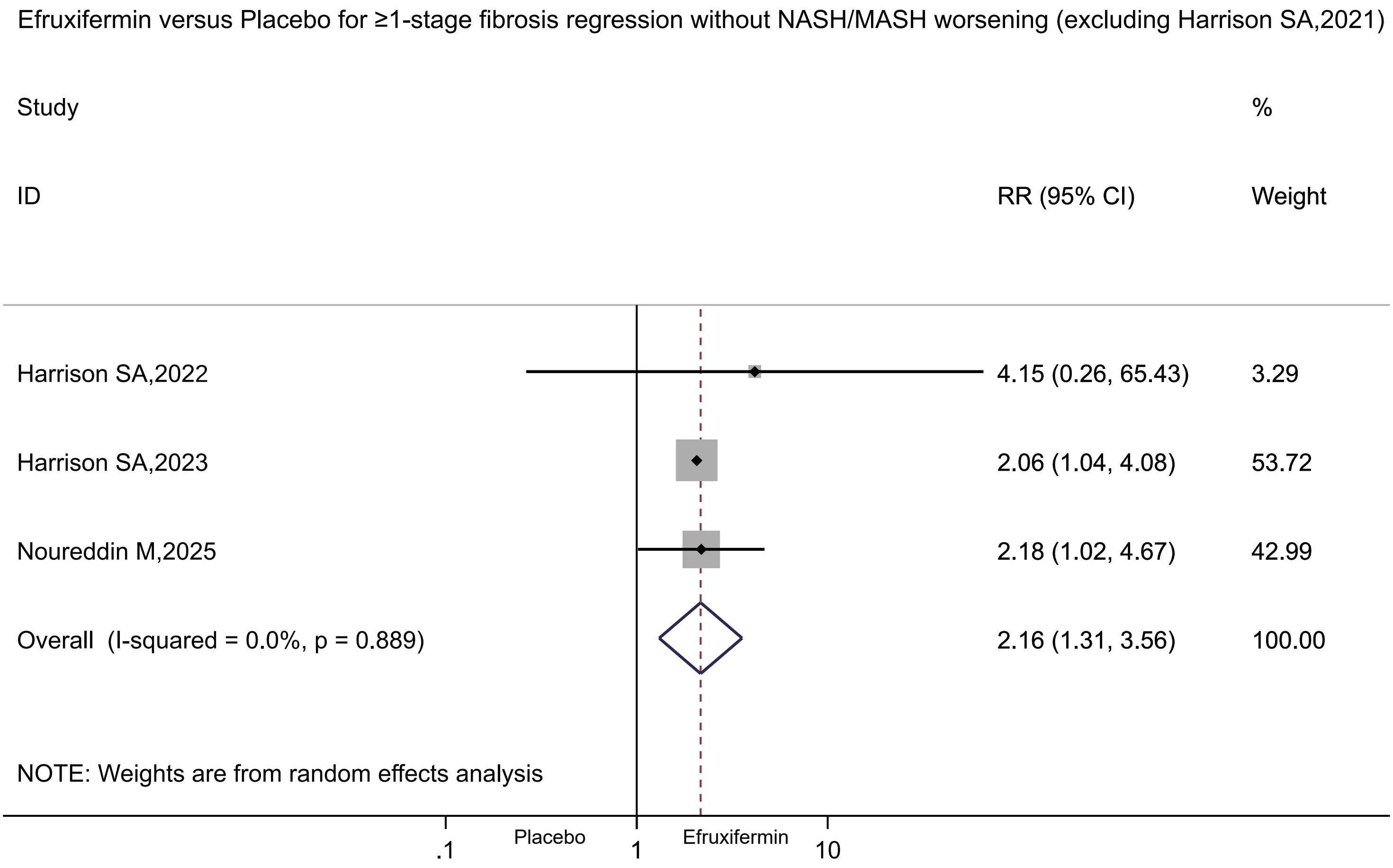

Figure S9. Sensitivity analysis: forest plot for meta-analysis comparing the effects of efruxifermin with placebo on fibrosis regression by at least one stage and no worsening in NASH/MASH, excluding Harrison SA,2021. NASH, Non-alcoholic steatohepatitis; MASH, Metabolic dysfunction-associated steatohepatitis; NAS, Nonalcoholic fatty liver disease activity score; RR, relative risk; CI, confidence interval.

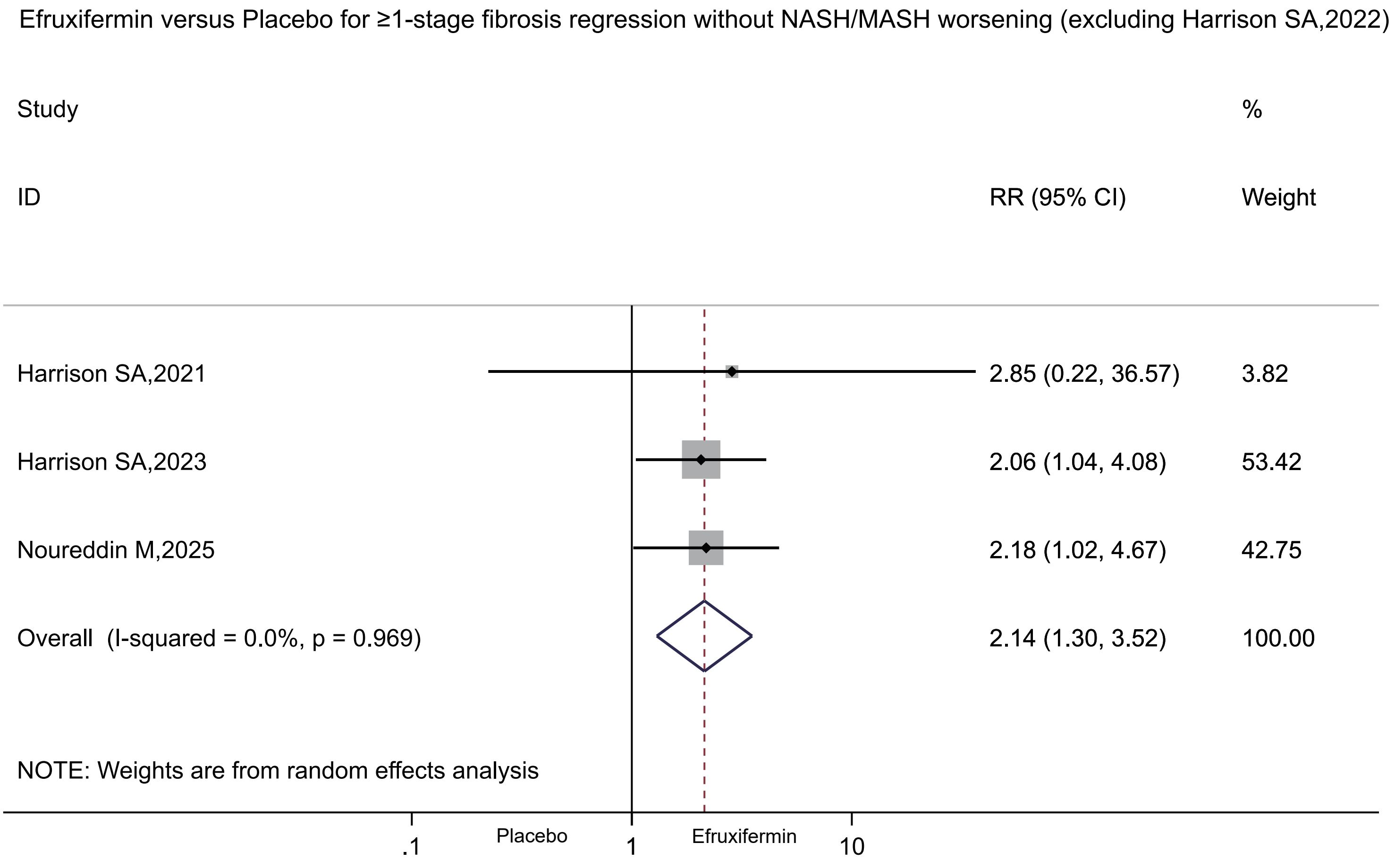

Figure S10 Sensitivity analysis: Forest plot for meta-analysis comparing the effects of efruxifermin with placebo on fibrosis regression by at least one stage and no worsening in NASH/MASH, excluding Harrison SA,2022. NASH, Non-alcoholic steatohepa-titis; MASH, Metabolic dysfunction-associated steatohepatitis; NAS, Nonalcoholic fatty liver disease activity score; RR, relative risk; CI, confidence interval.

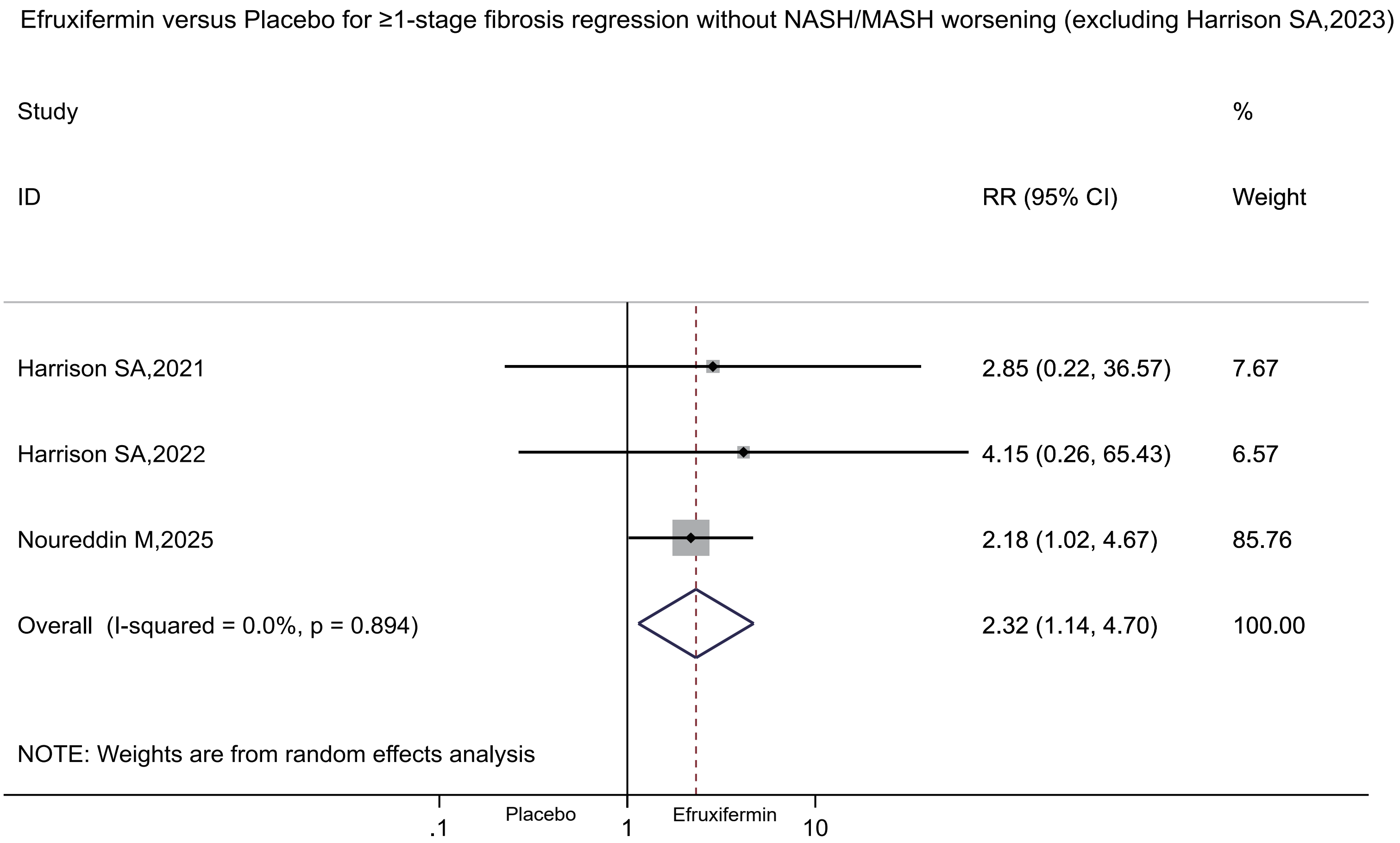

Figure S11. Sensitivity analysis: Forest plot for meta-analysis comparing the effects of efruxifermin with placebo on fibrosis regression by at least one stage and the absence of worsening in NASH/MASH, excluding Harrison SA,2023. NASH, Non-alcoholic steatohepatitis; MASH, Metabolic dysfunction-associated steatohepatitis; NAS, Nonalcoholic fatty liver disease activity score; RR, relative risk; CI, confidence interval.

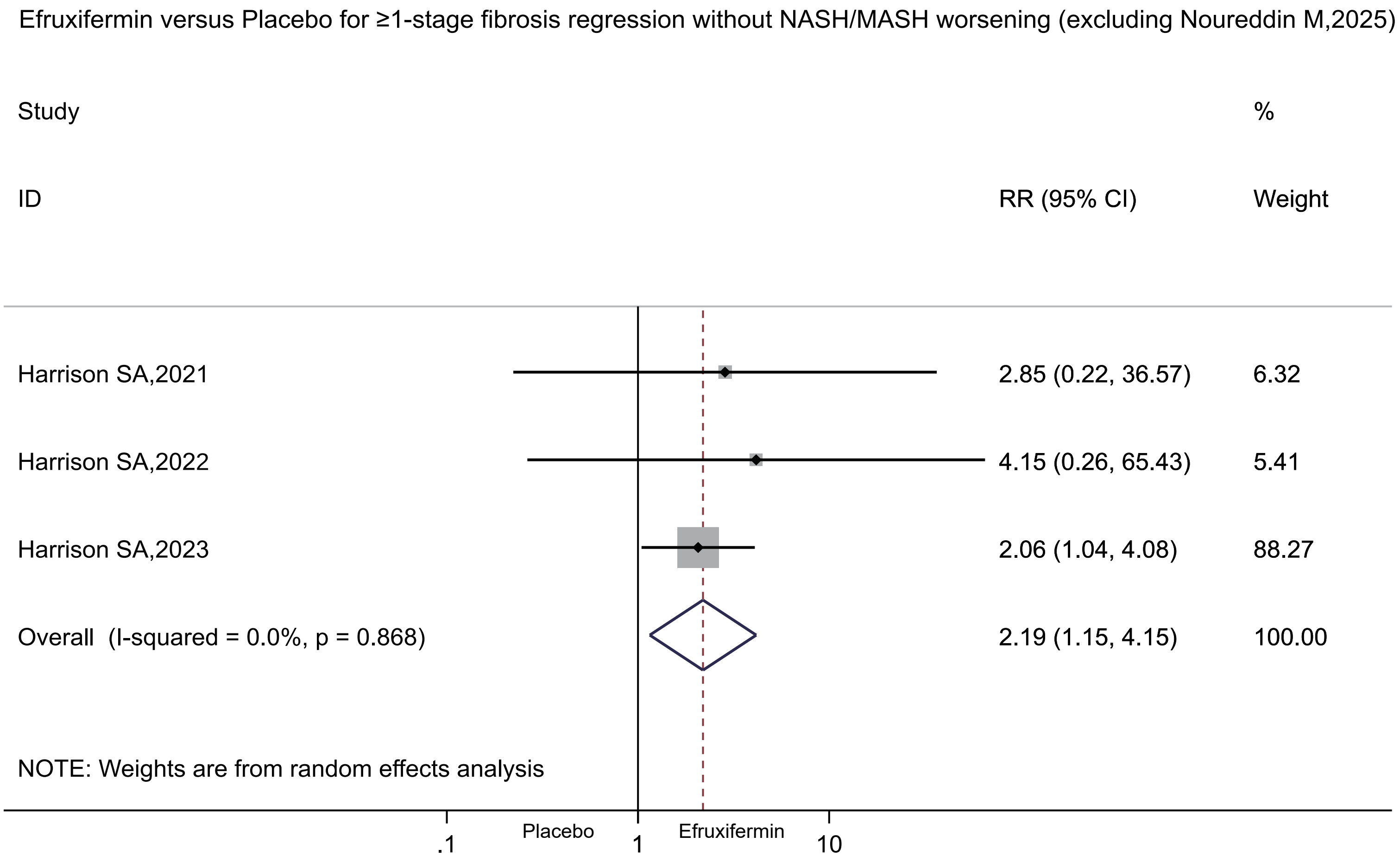

Figure S12. Sensitivity analysis: Forest plot for meta-analysis comparing the effects of efruxifermin with placebo on fibrosis regression by at least one stage and the absence of worsening in NASH/MASH, excluding Noureddin M,2025. NASH, Non-alcoholic steatohepatitis; MASH, Metabolic dysfunction-associated steatohepatitis; NAS, Nonalcoholic fatty liver disease activity score; RR, relative risk; CI, confidence interval.

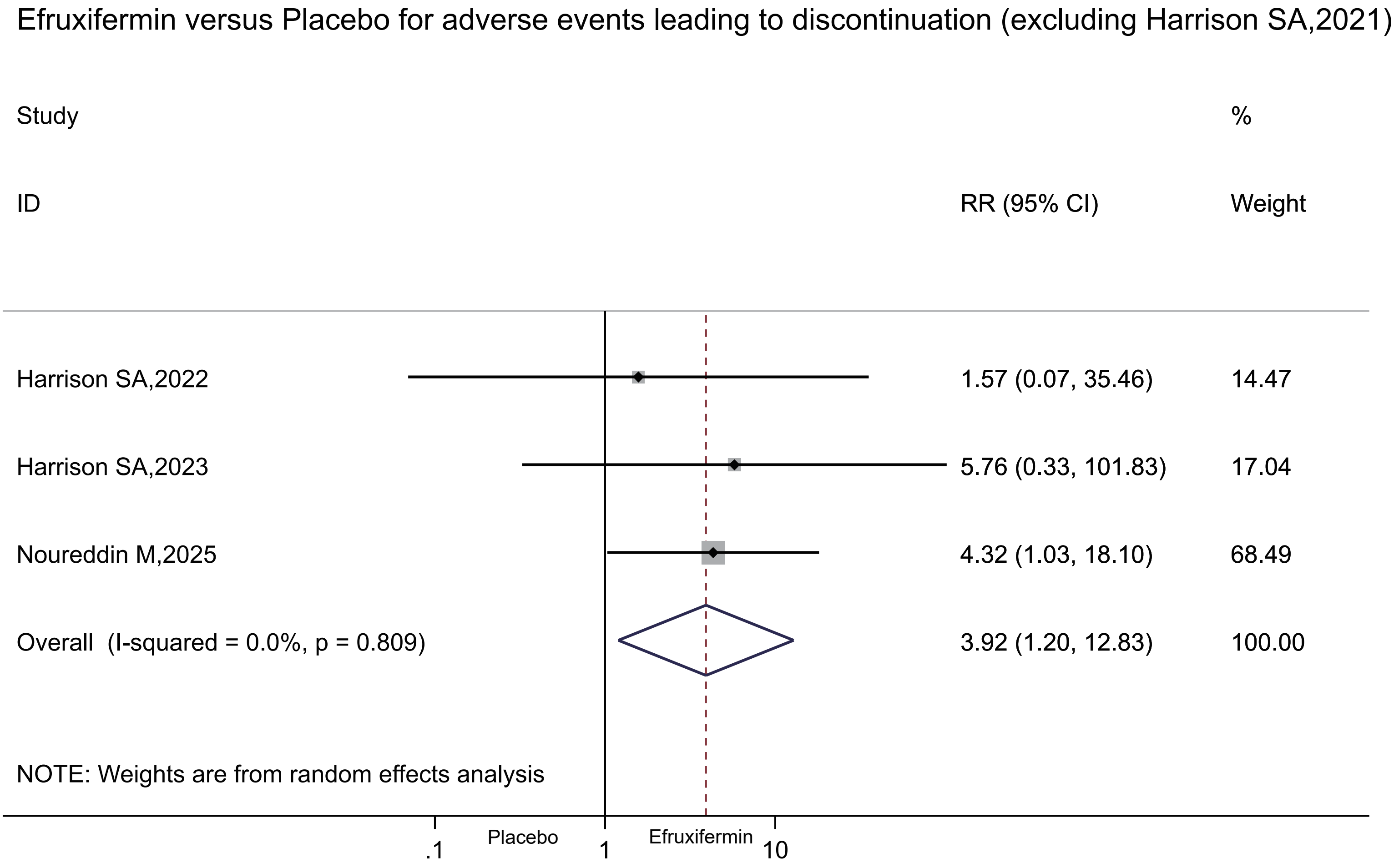

Figure S13. Sensitivity Analysis: Forest plot for meta-analysis comparing efruxifermin versus placebo on adverse events leading to treatment discontinuation, excluding Harrison SA, 2021. RR, relative risk; CI, confidence interval.

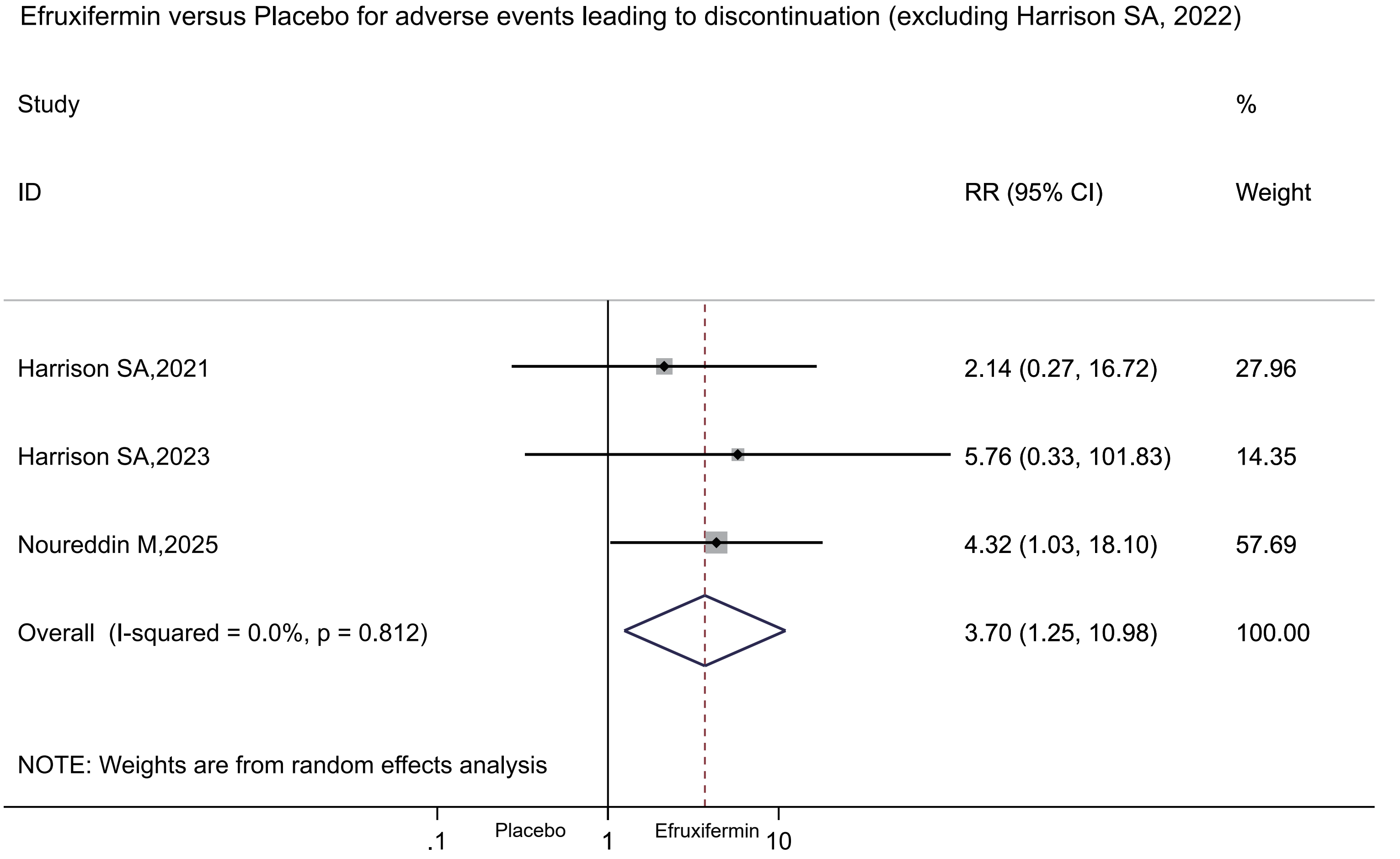

Figure S14. Sensitivity Analysis: Forest plot for meta-analysis comparing efruxifermin versus placebo on adverse events leading to treatment discontinuation, excluding Harrison SA, 2022. RR, relative risk; CI, confidence interval.

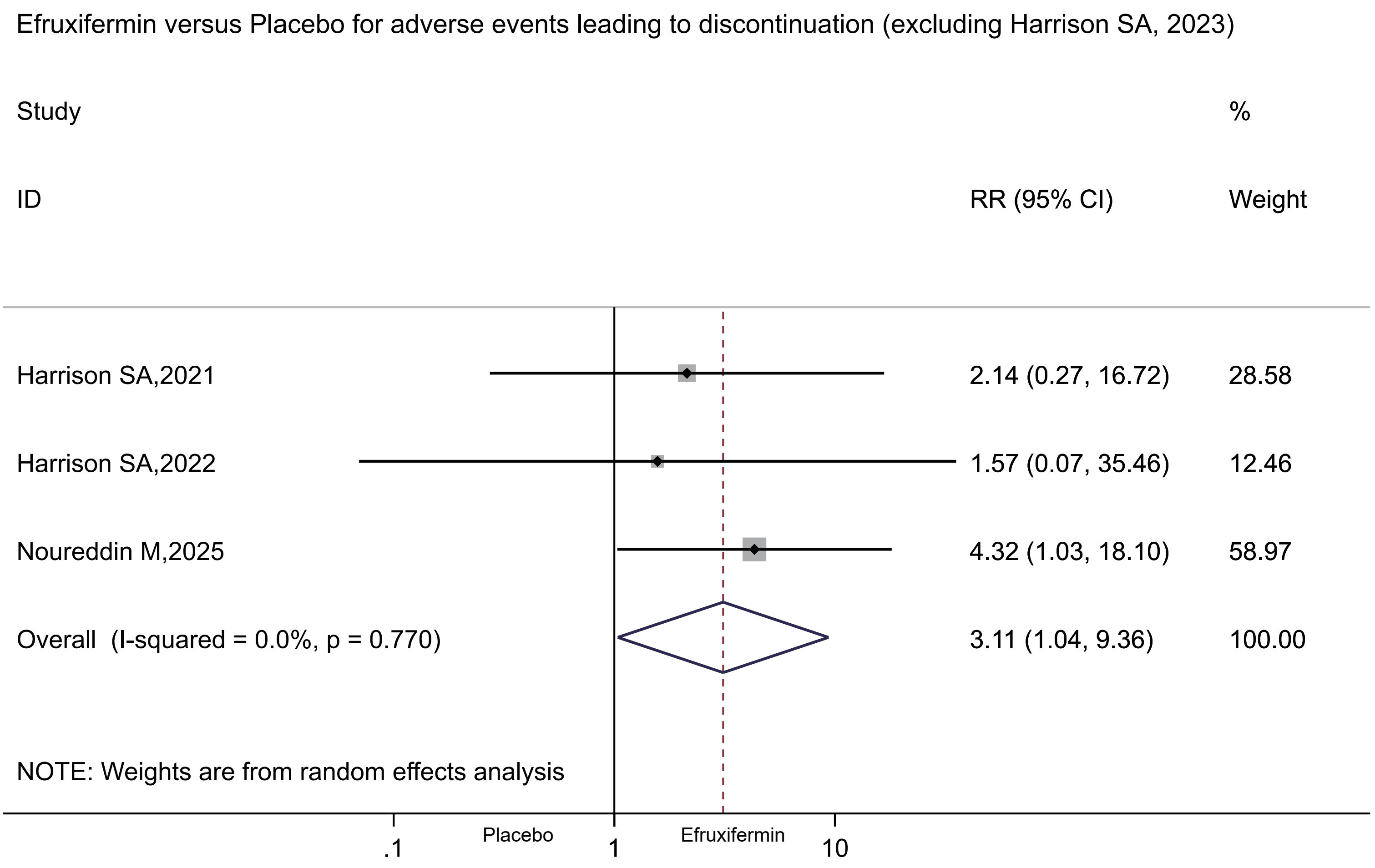

Figure S15. Sensitivity Analysis: Forest plot for meta-analysis comparing efruxifermin versus placebo on adverse events leading to treatment discontinuation, excluding Harrison SA, 2023. RR, relative risk; CI, confidence interval.

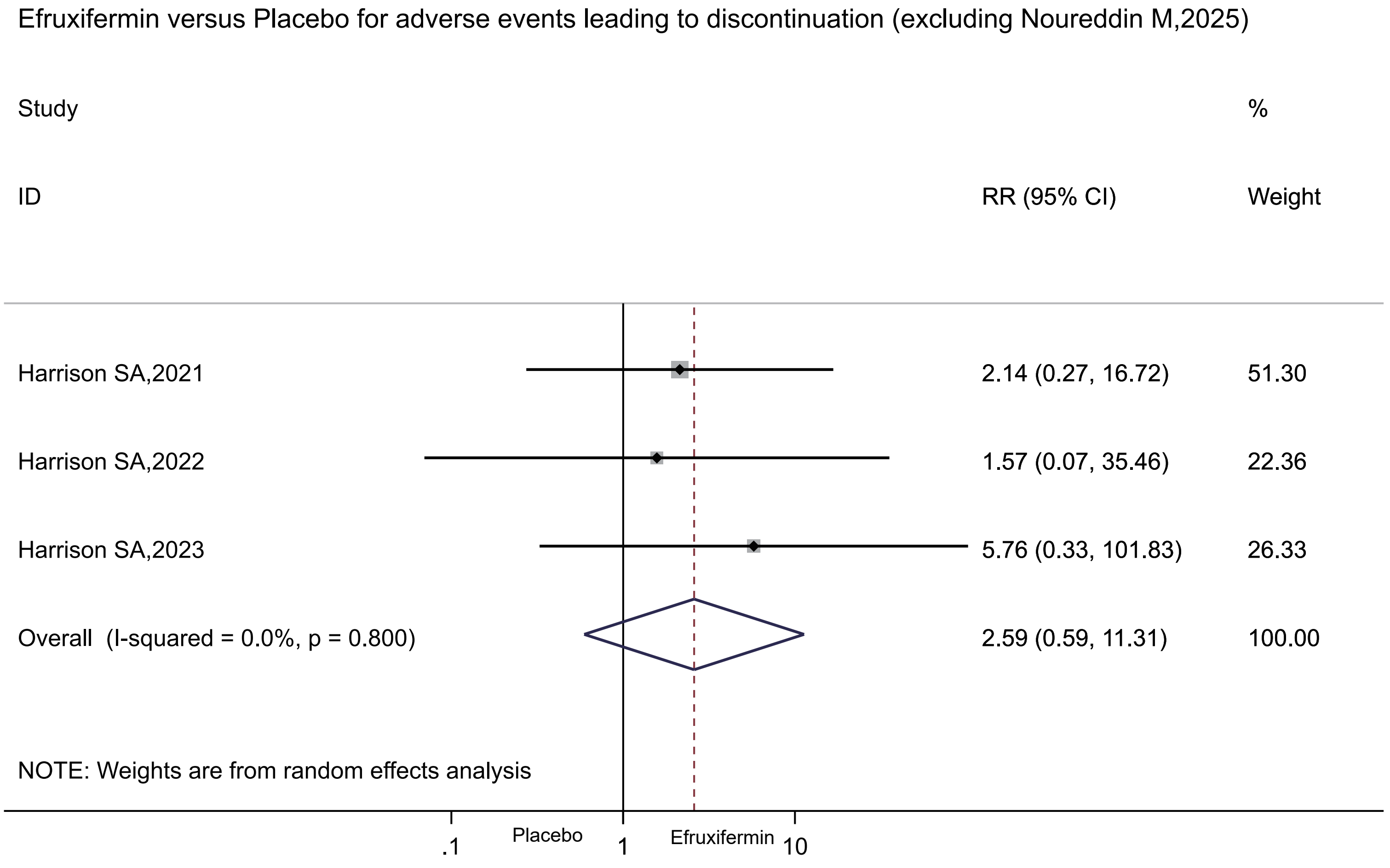

Figure S16. Sensitivity Analysis: Forest plot for meta-analysis comparing efruxifermin versus placebo on adverse events leading to treatment discontinuation, excluding Noureddin M,2025. RR, relative risk; CI, confidence interval.

Table S3 GRADE evidence profile.

Outcomes	No. of RCTs	Effect (95% CI)	Risk of Bias	Inconsistency	Indirectness	Imprecision	Publication Bias	Quality of evidence
Fibrosis regression by ≥1 stage and no worsening in steatohepatitis	4	RR: 2.18 (1.34, 3.57)	Serious[1]	No Serious	No Serious	No Serious	No Serious	Moderate
NASH/MASH resolution and improvement in fibrosis stage	2	RR: 5.15 (1.52, 17.47)	Serious[1]	No Serious	No Serious	Serious[2]	No Serious	low
Decrease in NAS≥2 with no worsening in fibrosis stage	3	RR: 3.34 (1.93, 5.80)	Serious[1]	No Serious	No Serious	Serious[3]	No Serious	low
Achieving a ≥2 stage improvement in fibrosis with no worsening of steatohepatitis	2	RR: 2.58 (0.72, 9.18)	Serious[1]	No Serious	No Serious	Serious[4]	No Serious	low
Absolute change in ELF test score	3	WMD: -0.66 (-0.82, -0.50)	No Serious	No Serious	No Serious	Serious[3]	No Serious	Moderate
Absolute change in LSM (kPa)	3	WMD: -2.66 (-4.3, -1.01)	No Serious	No Serious	No Serious	Serious[3]	No Serious	Moderate
Absolute change in Pro-C3 (μg/L)	2	WMD: -5.26 (-6.82, -3.71)	No Serious	No Serious	No Serious	Serious[3]	No Serious	Moderate
Percentage change in Pro-C3 (μg/L)	2	WMD: -23.48 (-37.37, -9.59)	No Serious	No Serious	No Serious	Serious[2]	No Serious	Moderate
Achieving ⩾30% reduction in HFF	2	RR: 4.69 (2.53, 8.71)	No Serious	No Serious	No Serious	Serious[3]	No Serious	Moderate
Achieving ⩾50% reduction in HFF	2	RR: 22.57 (5.78, 88.22)	No Serious	No Serious	No Serious	Serious[2]	No Serious	Moderate
Normalised liver fat (⩽5%)	2	RR: 13.03 (3.30, 51.50)	No Serious	No Serious	No Serious	Serious[2]	No Serious	Moderate
Absolute change in ALT (U/L)	3	WMD: -11.98 (-22.48, -1.49)	No Serious	Serious[5]	No Serious	Serious[2]	Not Serious	Low
TEAEs	4	RR: 1.06 (0.98, 1.15)	No Serious	No Serious	No Serious	Serious[4]	No Serious	Moderate

1. In the study by Harrison et al. (2021), there was a high risk of bias in the analyses related to histological endpoints due to the high rate of missing biopsy data and imbalance between groups.

2. Due to low total sample size and wide 95% CI.

3. Due to low total sample size.

4. For achieving a ≥2 stage improvement in fibrosis with no worsening of steatohepatitis and TEAEs the 95% CI includes the null effect.

5. High heterogeneity was observed for ALT.

Abbreviations: AE, adverse events; ALT, alanine aminotransferase; CI, confidence interval; ELF, enhanced liver fibrosis; LSM, liver stiffness measurement; MASH, metabolic dysfunction-associated steatohepatitis; NAFLD, non-alcoholic fatty liver disease; NAS, NAFLD activity score; NASH, non-alcoholic steatohepatitis; ProC3, N-terminal type-III collagen pro-peptide; RR, risk ratio; TEAEs, treatment-emergent adverse events; WMD, Weighted Mean Difference.
